# Supplementary material for: The impact of mask-wearing in mitigating the spread of COVID-19 during the early phases of the pandemic
Source: PLOS Glob Public Health. 2022 Sep 15;2(9):e0000954. doi: 10.1371/journal.pgph.0000954 (PMC10022328; doi:10.1371/journal.pgph.0000954)
Supplement: S1 Text — (DOCX) [file pgph.0000954.s001.docx]

**Supplementary Material for**

**Mask-Wearing During the COVID-19 Pandemic**

Ashwin Aravindakshan^1*^

Jörn Boehnke^*^

Ehsan Gholami^*^

Ashutosh Nayak^*^

^1^Corresponding Author Email: [aaravind@ucdavis.edu](mailto:aaravind@ucdavis.edu)

^*^University of California, Davis

**S1. Method**

This Section provides a detailed description of the reduced form econometrics model considered in our analysis. The model is derived from Susceptible-Infectious-Recovered (SIR) epidemiology models.

**S1.1 SIR Growth Rate Model**

Similar to the work by Hsiang et al [1], we employ a reduced form econometrics technique that relates the growth rate of active COVID-19 cases to the individual and institutional measures such as masks, social mobility, and governmental Non-Pharmaceutical Interventions (NPIs). The growth rate in econometrics is the first difference in the log of economic outputs in different periods. The growth rate model is a well-established method in econometrics where growth rates of economic output can be affected by different factors, e.g., policy. Similar to economic output, we model the growth rate of daily active cases and estimate how it is affected by wearing masks in public spaces, social mobility, and NPIs. The method also has roots in epidemiology models – SIR (Susceptible, Infectious, Recovered). We do not consider deaths and reinfection in this analysis.

Equations S1-S4 describe the SIR model where $S_{j,t}, I_{j,t},$ and $R_{j,t}$show the active susceptible, infectious, and recovered population at time $t$ in country $j$. $\beta_{j}$ is the rate of transmission and $\gamma_{j}$ is the rate of recovery in country $j$. Since we do not consider reinfection and deaths, $\gamma_{j}$ can be considered as the rate of removal from infectious population. $N_{j}$ is the total population of the country $j$. Equation S1 shows how infections spread from the infectious individuals to susceptible individuals. Equation S2 shows how infectious population changes over time as some susceptible individuals contract the disease while some already infectious individuals recover from the disease and test negative. Equation S3 shows how the number of recovered individuals increase over time as individuals recover after testing negative for the virus. Equation S4 is a feasibility constraint which ensures that the total population is accounted for in the model. Addition of Equations S1 – S3 yields Equation S4.

$$\frac{dS_{j,t}}{dt}=-\frac{\beta_{j}I_{j,t}S_{j,t}}{N_{j}} (S1)$$

$$\frac{dI_{j,t}}{dt}=\frac{\beta_{j}I_{j,t}S_{j,t}}{N_{j}}-\gamma_{j}I_{j,t} (S2)$$

$$\frac{dR_{j,t}}{dt}=\gamma_{j}I_{j,t} (S3)$$

$$\frac{dS_{j,t}}{dt}+\frac{dI_{j,t}}{dt}+\frac{dR_{j,t}}{dt}=0 (S4)$$

Since we model only the growth rate in the total confirmed cases, we consider Equation S2 in our analysis. Assuming $S_{j,t}\approx N_{j}$, we can rewrite Equation S2 as shown in Equation S5. It can be solved by integration as shown in Equation S6. If we consider daily growth rate ($t_{2}-t_{1}=1)$, Equation S6 can be simplified as shown in Equation S7, where $g_{j}$ is the growth rate and it is given by $\beta_{j}-\gamma_{j}$.

$$\frac{dI_{j,t}}{dt}=(\beta_{j}-\gamma_{j}{) I}_{j,t} (S5)$$

$$\int_{t_{1}}^{t_{2}} \frac{dI_{j,t}}{I_{j,t}}=\log\left( I_{j,t_{2}} \right)-\log\left( I_{j,t_{1}} \right)=\left( \beta_{j}-\gamma_{j} \right)(t_{2}-t_{1}) (S6)$$

$$\log\left( I_{j,t_{2}} \right)-\log\left( I_{j,t_{1}} \right)= g_{j} (S7)$$

Wearing face masks, reducing social mobility, and implementing of NPIs can alter the growth rate by changing $g_{j}$. We include country fixed effects (${country}_{j}$) to account for country specific heterogeneity in $g_{j}$. Equation S8 represents the growth rate model ($P$ is set of policies; $M$ is the set of indicators of social mobility, *W* is the set of weeks during the period of our analysis, and $J$ is the countries in our analysis). $mobility_{j,t,m}$ is the $m^{th}$indicator for social mobility, $week_{j,t,w}=1$ if day $t$ in country $j$ is in week $w$ after the initialization point for country $j$. To account for other factors, we consider several control variables (e.g., testing, Google Trends, fixed effect for week) as discussed in the next section.

$$g_{j,t+shift}=\theta_{0}+\theta_{c}.mask_{j,t}+\sum_{p\in P} \theta_{p}policy_{j,t,p}+\sum_{m\in M} \theta_{m}mobility_{j,t,m}+\theta_{e}.testing_{j,t}$$

$$+\sum_{w\in W} \theta_{w}week_{j,t,w}+\theta_{r}.trend_{j,t}+{\sum_{j\in J} \theta_{j}{country}_{j}+\epsilon}_{t} (S8)$$

We use government announcements on health resources, health monitoring, and tests conducted (per thousand individuals) to account for increased awareness and testing over time. We also use Google Trends on the keyword ‘*coronavirus’* to account for public self-awareness. We will discuss NPIs, Testing, and Google Trends later.

The econometrics approach of using the growth rate to estimate the effects of masks, social mobility, and NPIs has several advantages. The model can estimate the effect of the exogenous independent variables on the dependent outcome variable (growth rate). Since the right-hand side of Equation S5 can be empirically calculated, it does not explicitly require the knowledge of the relationship between exogenous variables and $I_{j,t}$. Thus, the model does not need to know the link between masks, NPIs, and social mobility on daily active cases (or cumulative confirmed cases) but can still estimate their effect on the growth rate of infectious cases. Using the growth rate, $I_{j,t}$can be estimated by integrating it from time 0 (or using previous integration up to the day $t-1$). Thus, this model is forward-looking.

The model is also able to handle underreporting in COVID-19. In the COVID-19 pandemic, data for an individual is recorded when only they are tested. Total confirmed cases (deaths and recovered cases) in publicly available datasets only provide information on the individuals who got themselves tested. Due to various reasons, e.g., lack of testing, lack of motivation to get tested, or lack of visible symptoms in symptomatic cases, it is being estimated that there is a massive underreporting in total confirmed positive cases. However, the growth rate model is agnostic to underreporting as it models the first difference in the log of confirmed cases. If the underreporting remains constant, we can multiply $I_{j,t}$ and $I_{j,t-1}$ with a constant over the considered time period, and it would not affect our estimation of growth rate (Equation S5).

Multiple studies have reported delays between the association of policies with COVID-19 spread [1]. This delay could be due to several reasons. One of the most commonly noted reasons is the incubation period (time between getting infected and onset of symptoms/knowing that individual is confirmed for COVID-19). During the incubation period, an individual may be asymptomatic. Incubation period is estimated to be 4 days to 14 days [2]. Another reason could be the testing time – the time it takes to get the confirmation of results. To model this delay, we use a lag variable. We use the cross-validation method to find the lag with the best fit for the data. We test and observe that the model performs best with a lag of 9 days.

**S1.2 Robustness and Control Function Models**

*Robustness Model*: As a robustness check to the growth rate model discussed above, we also use an exponential smoothing model to estimate the effect of masks, social mobility, and NPIs to validate the results from the base model in Equation S8. In this model, we use exponentially smoothed data for the right-hand side of Equation S8 for the past $w$ days, without using the $lag$ variable. Equation S9 shows this model where $<x>_{w}$ is exponentially smoothed over the last $w$ days. Smoothing function is shown in Equation S10. This method considers data in the recent future of day $t$instead of considering all the data leading up to the day$t$or data observed $lag$ days before as in the growth rate model in Equation S8. This model is analogous to counting the number of days a policy was active in the past $w$ days. We use exponential smoothing to include the lag effect of masks, NPIs and mobility on the growth rate.

$$\bar{g_{j,t+shift}}=\theta_{0}+{<\theta}_{c}.mask_{j,t}>_{w}+\sum_{p\in P} \theta_{p}<policy_{j,t,p>_{w}}+\sum_{m\in M} \theta_{m}<mobility_{j,t,m}>_{w}$$

$$+\theta_{e}<testing_{j,t}>_{w}+\sum_{w\in W} \theta_{w}week_{j,t,w}+\theta_{r}<trend_{j,t}>_{w}+{\sum_{j\in J} \theta_{j}{country}_{j}+\epsilon}_{t} (S9)$$

$$<x>_{w}=\frac{\sum_{l=1}^{w} {{0.8}^{w-l}x}_{t-w}}{\sum_{l=1}^{w} {0.8}^{w-l}} (S10)$$

Fig A shows the idea behind the exponentially smoothed model. In the growth rate model, we consider events that happened at a lag of $shift$ days. Thus, on day $t,$ we assign 0 weights to data from $t-shift+1$ to $t$. In the exponentially smoothed model, we assign exponentially reducing but non-zero weights to data for days $t-shift+1$ to $t$.


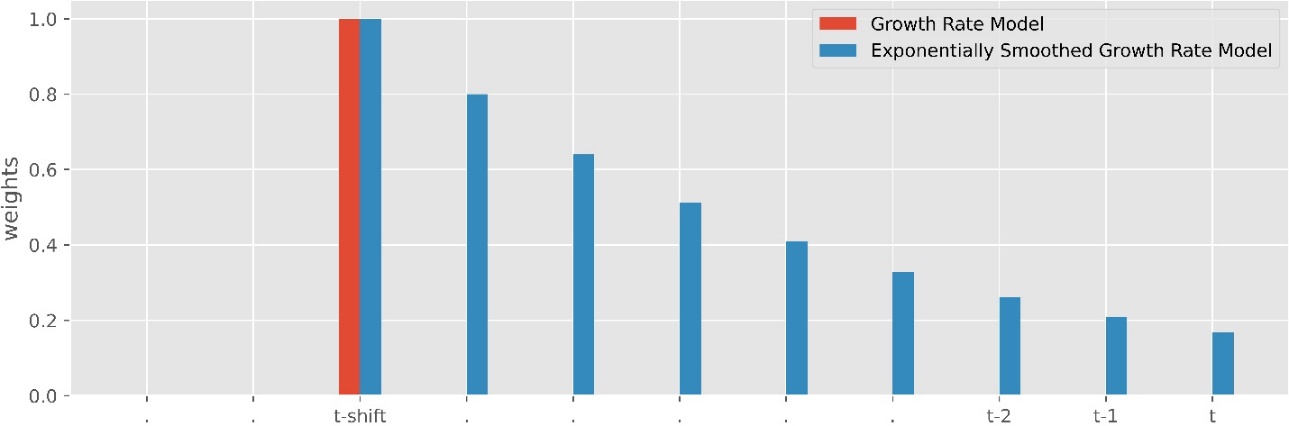


Fig A. Weights given to different data points for two models – growth rate model in Equation S8 (red) and exponentially smoothed growth rate model (blue) in Equation S9. Exponentially smoothed growth rate model considers all the data points in recent history to day $t$ instead of considering the events only on day $t-shift$. Weights decrease as we move closer to day $t$ to incorporate the delay in observing the effect of events in the recent future.

*Control Function Model*: We also consider a control function approach to check the robustness of the mask parameter from Equation S8. Countries have had different experiences with airborne diseases due to multiple outbreaks in the past, e.g., Severe Acute Respiratory Syndrome (SARS), Middle East Respiratory Syndrome Coronavirus (MERS-CoV) and H1N1 Influenza (Swine Flu). Countries with a severe outbreak of these airborne viruses were quick to adopt wearing face masks in public. This effect could potentially confound with the effect of the mask discussed in this paper. Therefore, we use a control function approach to isolate the effect of masks. Since the growth rate in COVID-19 is independent of the number of deaths per thousand people from SARS, MERS, and H1N1, it may affect the percentage of the population wearing mask, $mak_{j,t}$, but does not affect the growth rate. Thus, we use deaths from previous diseases as a control function. Fig B shows the total deaths per thousand people for different countries.


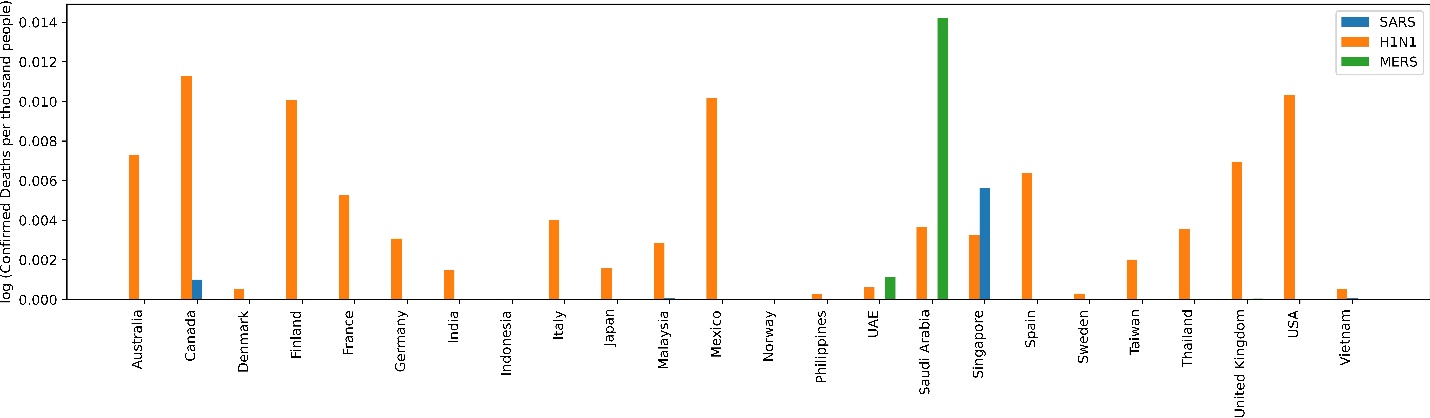


Fig B. Logarithm of number of confirmed deaths with SARS, H1N1 and MERS-CoV per thousand people across 24 countries considered in this study.

In the control function model, we first predict average mask wearing in country *j* $\hat{mask_{j}}$ using the number of deaths from SARS, H1N1 and MERS in country $j$ as covariates in ordinary least square linear regression estimation. We use $d_{j,dis}$ as predictor variable where $dis\in\left\{ SARS, H1N1, MERS \right\}$and $d_{j,dis}$is the number of deaths per thousand people in country $j$ from disease $dis$. Specifically we use $\hat{d_{j,dis}}=1 if d_{j,dis}>median(d_{dis}), and 0 otherwise$, where ${median(d}_{dis})$ is median among countries for a specific disease $dis$. The model to predict $mask_{j,t}$ is shown in Equation S11. After estimating $\hat{mask_{j}}$, we use the error $mask_{j,t}-\hat{mask_{j}}$ in Equation S9 as a covariate. Control function model is shown in Equation S12.

$$\hat{mask_{j}}=d_{j,SARS}+d_{jH1N1}+d_{j,MERS}+\epsilon_{m} (S11)$$

$$g_{j,t+shift}=\theta_{0}+\theta_{c}.mask_{j,t}+\sum_{p\in P} \theta_{p}policy_{j,t,p}+\sum_{m\in M} \theta_{m}mobility_{j,t,m}+\theta_{e}.testing_{j,t}$$

$$+\sum_{w\in W} \theta_{w}week_{j,t,w}+\theta_{r}.trend_{j,t}+{\sum_{j\in J} \theta_{j}{country}_{j}+\theta_{md}(mask_{j,t}-\hat{mask_{j})}+\epsilon}_{t} (S12)$$

Wearing face masks in public is common in many Asian countries, as compared to countries in Europe or America [3, 4]. One of the reasons is their recent experience with airborne diseases. Another reason could be air pollution or the culture of wearing face masks. We do not account for the different trends in wearing face masks among countries due to pollution or culture. However, we believe the country-fixed effects could capture the country-wise trends in wearing face masks.

**S2. Data Collection and Processing**

We model the effect of wearing face masks, change in social mobility, and government enforced Non-Pharmaceutical Interventions (NPIs) in infecton growth rate. We select the countries with a publicly available dataset for wearing face masks and community mobility. We collect data from February 21, 2020, to July 8, 2020. This Section discusses the different datasets used in our analysis to isolate the effect of masks, social mobility, and NPIs in containing the spread of the contagious SARS-CoV-2 (COVID-19) virus.

**S2.1 Masks**

We collect mask data from surveys conducted by YouGov [5]. YouGov is an international internet-based market research company specializing in opinion polls through online methods. YouGov used online surveys as their COVID-19 behavior change tracker. They conducted surveys periodically in some countries of the world to estimate the propensity of the percentage of people that wear face masks when they go out in public spaces. These surveys were conducted every week. Fig C shows the raw survey numbers from YouGov. As the surveys were conducted periodically and not every day, we used linear interpolation to estimate the percentage of the population that wear face masks in public spaces (Fig D). Note that the online survey does not include data for type (quality) of masks or how people wear masks (insufficient quality or incorrect method of covering face masks, e.g., touching the surface, not covering nose or mouth - might not be effective in controlling the spread of virus). Thus, our estimation of mask effects in this work would be an estimation of the behavior of wearing masks. In our analysis, we normalize the number for mask wearing such that $0\leq mask_{jt}\leq1, \forall j, \forall t$.


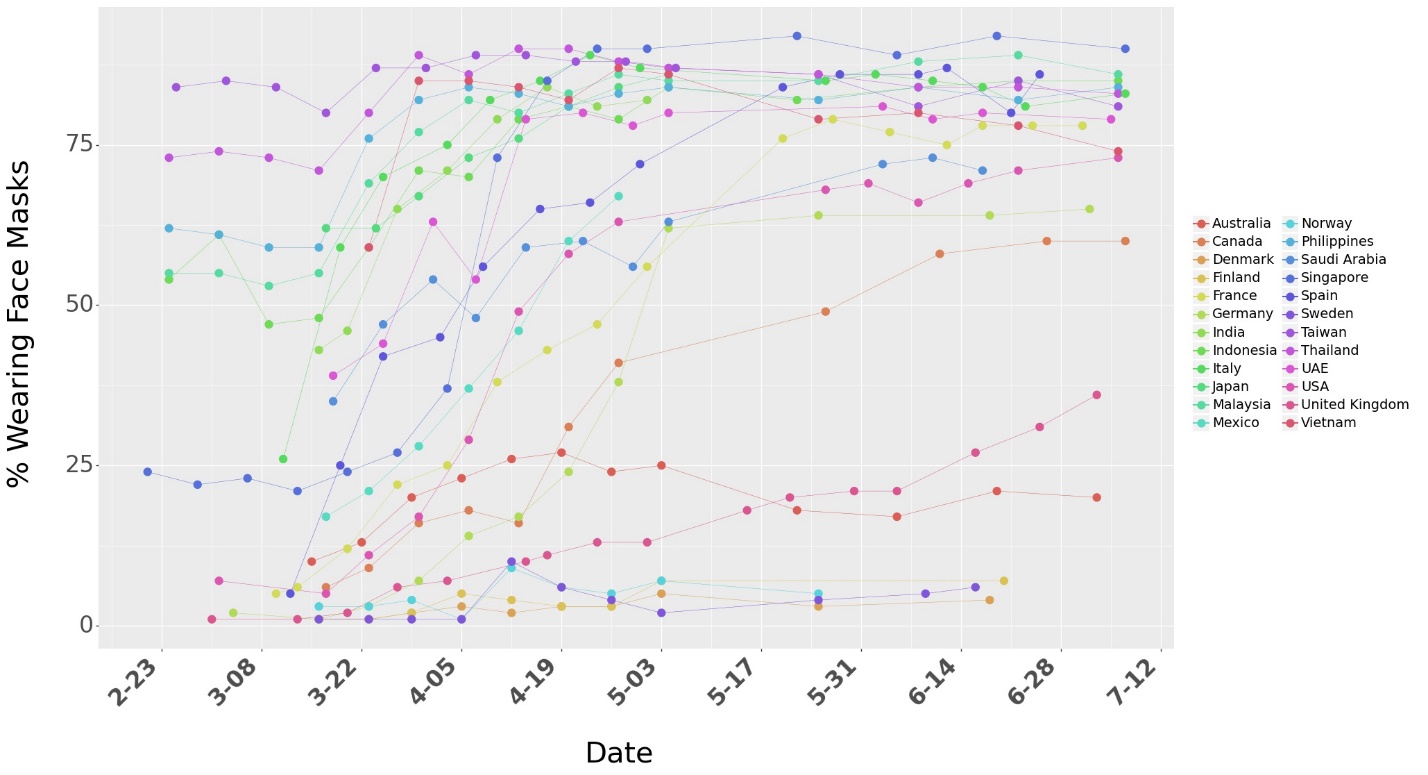


Fig C. Raw data for surveys on percentage of people who say they wear a face mask when in public spaces. The dots represent the raw numbers from the survey data from YouGov. The lines are shown for better visualization.


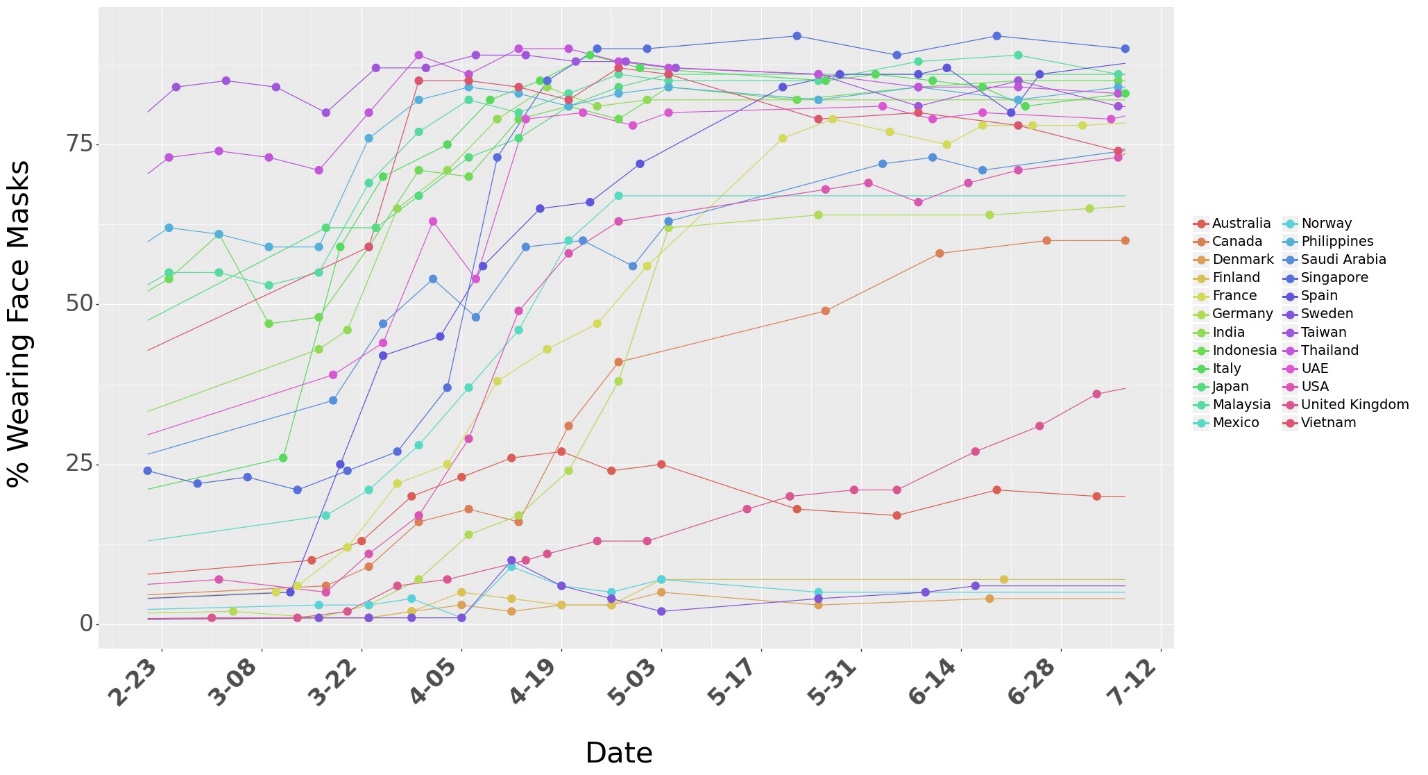


Fig D. Survey data on percentage of people who say they wear a face mask when in public spaces. We use linear interpolation to consider mask numbers for days between surveys days. The dots represent the raw numbers from surveys.

**S2.2 Active Cases**

We use the timeline for total confirmed cases, and total recovered cases from Johns Hopkins Coronavirus Research Center [6] to find the daily active cases across different countries. We use the daily active cases to calculate the outcome variable of our model - growth rate. Fig E shows the cumulative confirmed cases, cumulative recovered cases, and daily active cases for different countries. We use a 7-day moving average for daily cumulative confirmed cases and cumulative recovered cases.


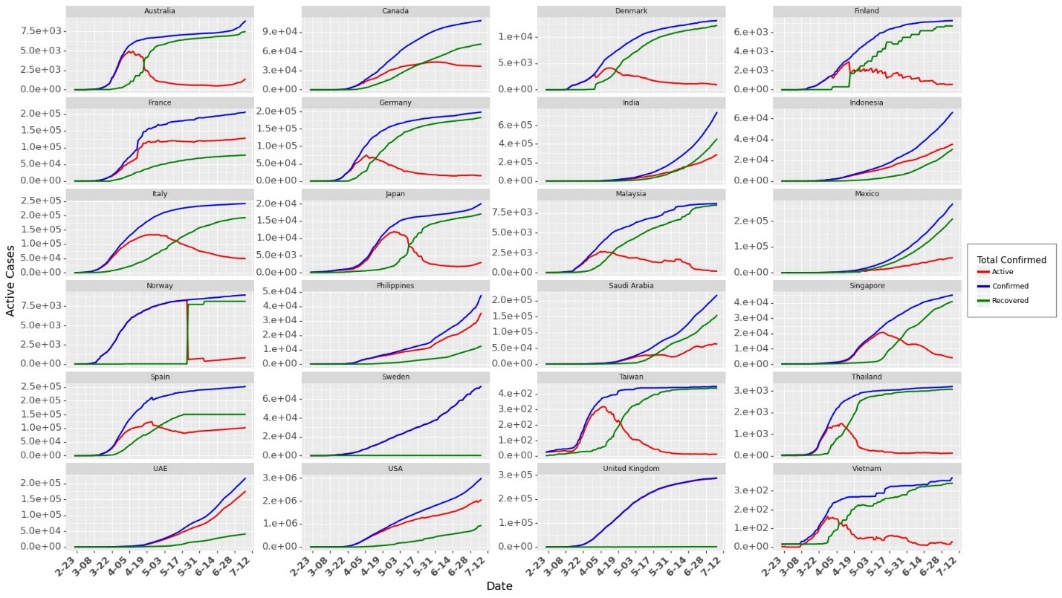


Fig E. Cumulative confirmed cases, cumulative recovered cases and daily active cases across 24 countries. We observe data reporting issues in Norway, Sweden and United Kingdom. We use cumulative confirmed cases for calculating the growth rates for these countries.

**S2.3 Growth Rate**

We use daily active cases to estimate the daily growth rate for these countries. Growth Rate (Equation S7) can be very volatile at the start of the pandemic due to the low number of cases in the early stages. For example, a unit increase in $I_{j,t}$ will record a growth rate of 0.4 when $I_{j,t}=2$ as compared to a growth rate of 0.0004 when $I_{j,t}=1000$ (growth rate is calculated as the first difference in log of active cases in consecutive days). Similarly, during the later stages of the pandemic (at least when the first wave is slowed down for some countries), the growth rate could be affected by multiple other factors such as awareness or changed individual behavior. To avoid these issues, we use the data for the first 60 days for a country (after we start collecting data for a country following the ‘*th*’ – will be discussed shortly).

Unlike Hsiang et al. [1], we use data for 60 days and do not restrict to the initial phase when the cases rise exponentially. In §Robustness check, we discuss the model’s performance (and changes in model parameter estimates) as we add more/fewer data in the model from 24 countries. To filter out the volatile growth rate during the start of the pandemic, we consider data for each country when the daily new cases cross a threshold $th.$We define this threshold as the day when the seven-day average of daily new cases in a country crosses $th=$20% of the peak case observed in that country (till July 8, 2020). We select $th$based on the maximum likelihood estimate of the growth rate model (mechanism for selecting $th$ is discussed later in the §Robustness Check section). Fig F shows the daily new cases, and Fig G shows the respective growth rate. We use data for a maximum of 60 days for a country, from the day its daily cases cross the threshold. Thus, our dataset contains an unbalanced panel data from 24 countries.


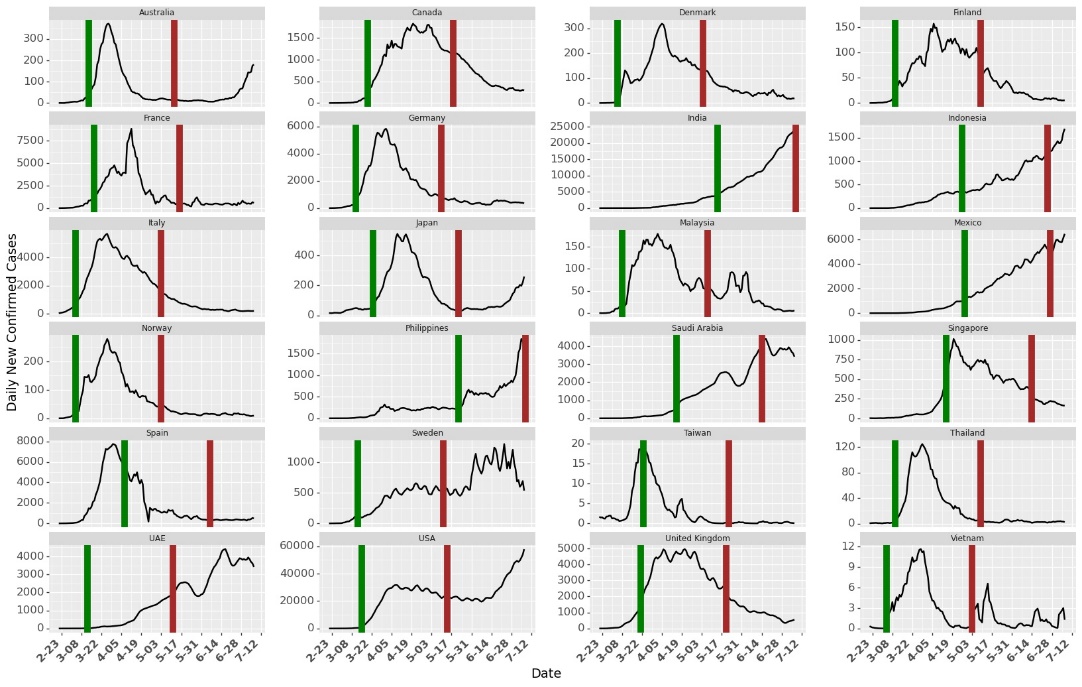


Fig F. Daily New Cases. The green line marks the day when daily new cases in that country crossed the threshold. Brown line shows the end of 60 days of data collected for each country. For countries where cases are still increasing vis-à-vis India and Philippines, we collected fewer data points than 60 days.


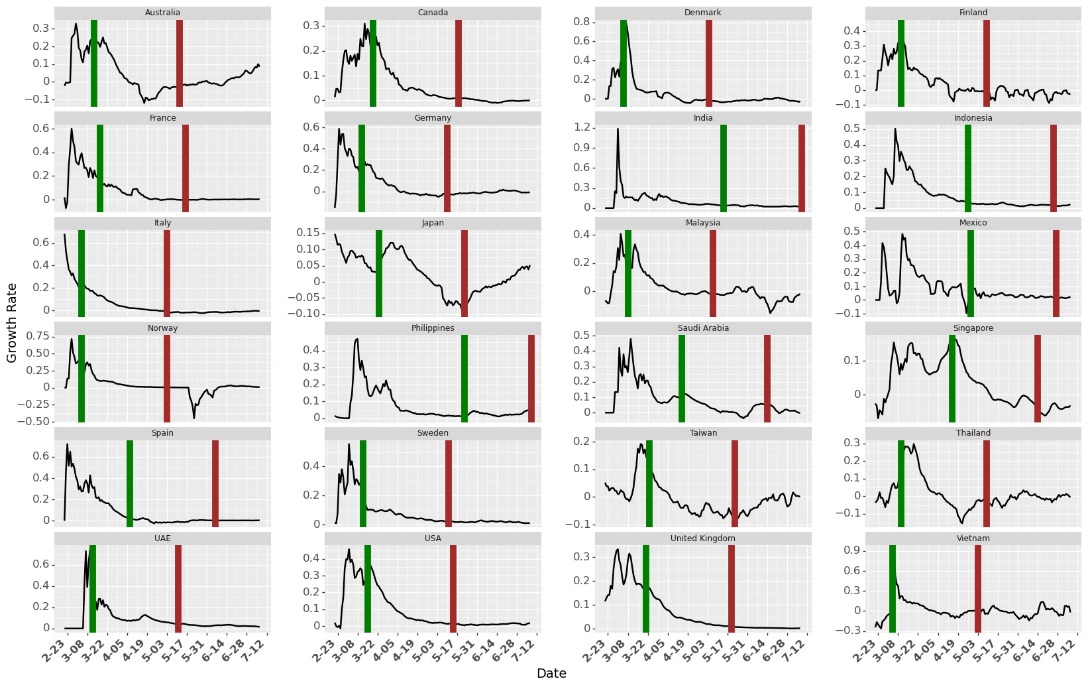


Fig G. Growth rate across Countries. The green line marks the day when daily new cases in that country crossed the threshold. Brown line shows the end of 60 days of data collected for each country. We use cumulative confirmed cases to calculate growth rate for Norway, Sweden and United Kingdom. Collecting data after the green line allows us to filter initial noisy growth rate from that country.

**S2.4 Community Mobility**

Google’s COVID-19 Community Mobility Reports [7] provides information on how movement trends change over time across different types of locations in different countries. The mobility numbers are calculated based on the change in trend from the baseline (details in the report on how Google calculates the baseline). The report tracks movement trends over time by geography across different categories of places such as retail and recreation, groceries and pharmacies, parks, transit stations, workplaces, and residential. Fig H shows the community mobility across different countries.

We observed a high correlation between the social mobility numbers from Google across different types of locations. This may lead to unstable parameter estimates due to multicollinearity in parameter estimation using ordinary least squares. Based on the correlations, we consider the mobility in Parks and Transit stations as our measure for mobility. We also confirm these two categories using a Lasso regression (more details in Section §Robustness Check in Section 4.5.3). The Lasso regression model pushes the coefficients of correlated variables (variables that do not add much information to the model) to 0 and gives non-zero weights to only two mobilities: Parks and Transit stations.

Table A shows the correlation matrix between the mobility across different locations. The correlation matrix shows that transit stations is highly correlated with mobility in Retail and Recreation, Grocery and Pharmacy, and Residential. Mobility in transit stations is negatively correlated with mobility in Residential as - fewer people travel more people are staying home. Thus, mobility in transit stations can capture the information from mobility across all other locations except Parks. Henceforth, we include mobility in Parks and Transit stations as a measure of mobility (as also selected by the Lasso Regression model). In our analysis, we normalize the number for social mobility such that $0\leq mobility_{j,t,m}\leq1 \forall j, \forall t,\forall m$.

Table A. Correlation Matrix for Community Mobility in Different Locations from Google and Apple.

|  | **Retail and Recreation** | **Grocery and Pharmacy** | **Parks** | **Transit Stations** | **Workplace** | **Residential** | **Driving** | **Walking** |
| --- | --- | --- | --- | --- | --- | --- | --- | --- |
| **Retail and Recreation** | 1.00 | 0.85 | **0.53** | **0.90** | 0.75 | -0.85 | 0.81 | 0.82 |
| **Grocery and Pharmacy** | 0.85 | 1.00 | **0.45** | **0.78** | 0.67 | -0.74 | 0.69 | 0.67 |
| **Parks** | **0.53** | **0.45** | **1.00** | **0.42** | **0.16** | **-0.51** | **0.73** | **0.67** |
| **Transit Stations** | **0.90** | **0.78** | **0.42** | **1.00** | **0.84** | **-0.89** | **0.73** | **0.78** |
| **Workplace** | 0.75 | 0.67 | **0.16** | **0.84** | 1.00 | -0.87 | 0.53 | 0.58 |
| **Residential** | -0.85 | -0.74 | **-0.51** | **-0.89** | -0.87 | 1.00 | -0.74 | -0.75 |
| **Driving** | 0.81 | 0.69 | **0.73** | **0.73** | 0.53 | -0.74 | 1.00 | 0.91 |
| **Walking** | 0.82 | 0.67 | **0.67** | **0.78** | 0.58 | -0.75 | 0.91 | 1.00 |

Table B shows the summary statistics for the community mobility.

Table B. Summary Statistics on Mobility Trends from Google.

| Mobility | Min | Mean | Max | Std Dev | 25^th^ percentile | 75^th^ percentile |
| --- | --- | --- | --- | --- | --- | --- |
| Retail and Recreation | -96 % | -32.3 % | 23 % | 27.1 % | -53 % | -10 % |
| Grocery and Pharmacy | -94 % | -10.1 % | 51 % | 18.7 % | -19 % | 2 % |
| Parks | -91 % | 4.7 % | 517 % | 65.5 % | -38 % | 24 % |
| Transit Stations | -92 % | -38.8 % | 14 % | 23.9 % | -57 % | -20 % |
| Workplaces | -90 % | -27.7 % | 57 % | 23.8 % | -45 % | -7 % |
| Residential | -13 % | 12.8 % | 55 % | 10.4 % | 4 % | 19 % |


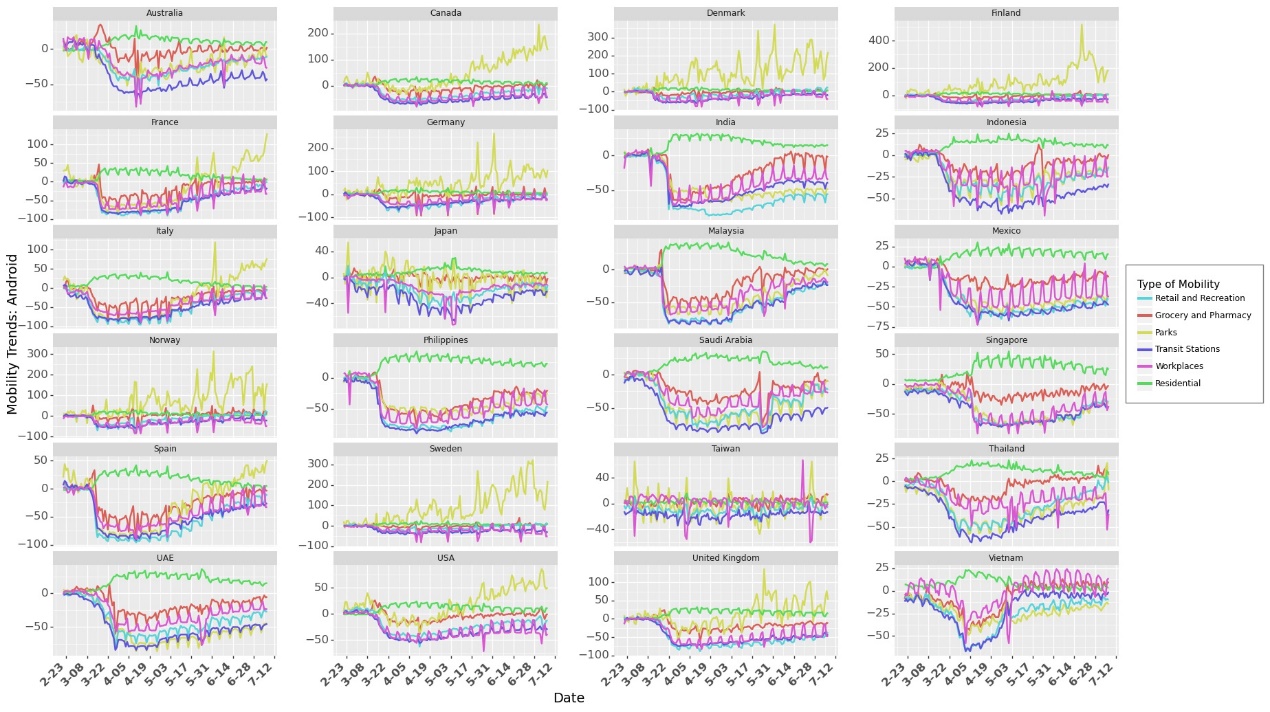


Fig H. Community Mobility Trend from Google using Android Operating System in different countries.

Google community mobility reports use data from users with the android operating system. Also, they collect data from users who allow location sharing (Google does not disclose any personal information in Community Reports). To check the robustness of the model and our estimates on the effect of mobility, we also use mobility data from Apple Mobility Trend reports [8] to validate the results from Google Community Mobility reports. The Apple Mobility reports show a relative volume of direction requests per country/region, sub-region, or city compared to a baseline volume on January 13, 2020. Apple compares the relative volume for Driving, Transit, and Walking in their dataset. However, we could not find the data on Transit for all the 24 countries considered in this paper. So, we use the timeline for Driving and Walking as a proxy for measuring social mobility. Table C provides the summary statistics on Apple Mobility Trends. Fig I illustrates the mobility trends for Driving and Walking across 24 countries.

Table C. Summary Statistics on Mobility Trends from Apple.

| Mobility | Min | Mean | Max | Std Dev | 25^th^ percentile | 75^th^ percentile |
| --- | --- | --- | --- | --- | --- | --- |
| Driving | -100 % | -28.2 % | 184 .9% | 48.5 % | -64.9 % | -6.2% |
| Walking | -100 % | -35.7 % | 94.4 % | 44.1 % | -72 % | 0.58 % |


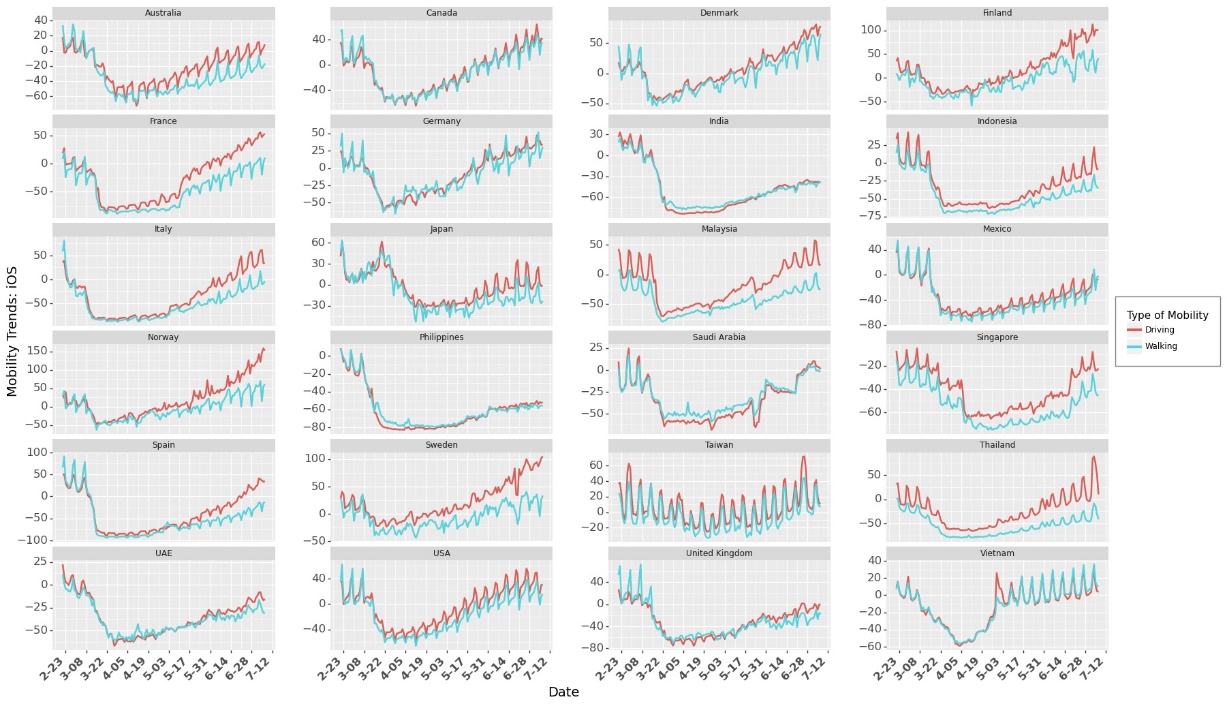


Fig I. Community Mobility Trend from Apple using iOS in different countries.

**S2.5 Non-Pharmaceutical Interventions (NPIs)**

Governments (and thier policies) play a critical role in fighting a pandemic. Vaccines may take a long time to be available, particularly for a new disease, e.g., COVID-19. In an ongoing pandemic, we cannot depend only on vaccines but need vital government interventions (institutional measures) to control the spread of the disease. During such times, governments must take various measures, e.g., increasing testing infrastructure to control the spread of infections. These NPIs help decrease mobility (for example, travel bans imposed restrictions on travel across states/regions/countries while it also helped restrict mass gatherings in places such as transit stations. Since COVID-19 spreads through person-to-person physical interaction (or prolonged proximity), governments introduced various policies (Non-Pharmaceutical Interventions or NPIs) for social distancing to minimize person-to-person interaction. They also introduced closures of places where people gather together simultaneously, e.g., schools or businesses. However, these government policies seriously affected businesses [9], leading to economic shutdowns which adversely affected the poor community [10]. The effect of these shutdowns may also lead to prolonged economic hardships, e.g., closure of some businesses and employment [11].

Since these policies directly affect the livelihood of a majority of the population across the world, it is crucial to investigate their impact on controlling the spread of the disease. As these policies were implemented at different times across different countries, it allows us to explore the combined effect of these policies. Estimating the combined effect of these policies could help the governments in future (or current) pandemics to introduce effective policies and may not necessarily lead to complete lockdown unless extremely necessary. Please note that some of these policies were introduced simultaneously, or some were implemented first, and some were implemented consistently after some other policies, we do not claim any causal effect of the policy on the growth rate. It is difficult to isolate the effect of individual policies as the implementation of policies was not randomly sequenced across countries.

We use Coronanet dataset from Cheng at al [12] for NPIs. They collected information on all the government policies introduced by different countries worldwide. They categorized the policies into 19 different *policy_types*. We use their categorization to build our model. The policies were implemented at different levels – National, Provincial and Municipal. This work considers the policies implemented at the National and Provincial levels. From February 21, 2020, to July 8, 2020, we check if a policy $p$ was implemented in a country $j$or not on the day $t$. If the policy was implemented, we assign a value of 1 to $s_{j,t,p}.$ If the policy was introduced at a provincial level (could be introduced by the central government or a respective state government), we increase $s_{j,t,p}$ using the population of the state. Equation S13 explains $s_{j,t,p}$ if a policy $p$ is employed at a provincial level where $N_{j,s}$the population of state $s$ in country $j$. After identifying $s_{j,t,p}$for all countries over the period of our analysis (considering all the entries in the dataset), we use normalization using maximum value in a country such that $0\leq s_{j,t,p}\leq1 \forall j, \forall t, \forall p$ as shown in Equation S14.

$$s_{j,t,p}= s_{j,t,p}+\frac{N_{j,s}}{N_{j,s}+N_{j}} (S13)$$

$$s_{j,t,p}= \frac{s_{j,t,p}}{\max_{t,p} s_{j,t,p}} (S14)$$

The dataset contains 5,816 entries on policies (some of the policies were announcements/recommendations/new entry or an update to existing policy) at National and Provincial level. The dataset provides detailed information on the type of the data entry (e.g. policy type, description of the policy). The statistics on the types of policies is shown in Fig J. Fig J also provides a count of entries of each policy type. The data set contains 20 $policy\_types.$ Fig J shows how many countries implemented (light grey bars) a particular $policy\_type$. It also shows how many countries implemented a particular $policy\_type$ at national or provincial level (light blue bars and dark blue bars respectively). The dark grey bars show the total number of entries (divided by 100 for visualization) for all $policy\_types$.


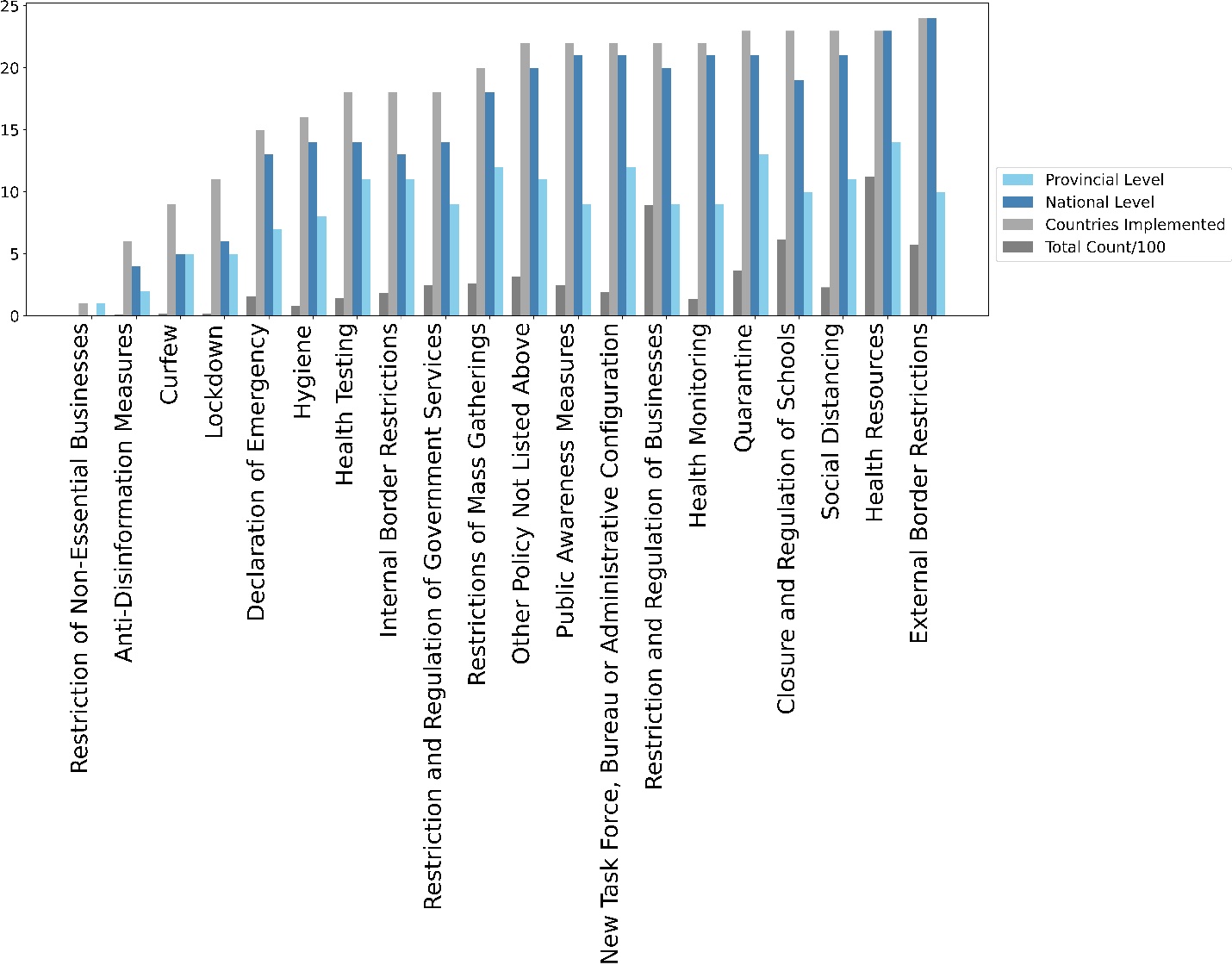


Fig J. Policy Implementation at National and Provincial Levels Across Different Countries

We use the text description of the policy to identify if an entry was an update, recommendation, or actual implementation. If the entry was an announcement or an update for a policy with a start date and an end date, we give a weight of 0 to that entry in the dataset because if there is a policy update, it could recount that policy. The policies could have been implemented differently across different countries, even if they were categorized in the same policy type. For example, a country may impose Social distancing rules from 4 pm – 8 pm while another may impose them from 6 am – 6 pm. It may differ across different states in the country. However, we do not consider the variations in the implementations of policies in this research.

As we have survey numbers for wearing face masks at a national level, we consider policy types implemented across most countries. Therefore, we do not consider Anti-disinformation measures, Curfew and Lockdowns. Curfew and Lockdowns are similar to Quarantine and Restrictions of Mass Gatherings (which lead to the closure of places of mass gatherings) so we can ignore them for the purpose of this research. Moreover, Curfew and Lockdowns affect the community mobility, which can be accounted for by social mobility trends from Google Community Mobility Reports (discussed in the previous section).

We also do not consider Hygiene Announcements and New Task Force policy as these were administrative announcements and did not have much effect on the growth rate of the infection. Some policies did not have a start date and end date. We calculate the cumulative number of times announcements were made for such categories. Health Testing, Health Monitoring, and Health Resources are administrative announcements, so we combine them into one Health Resources policy. Using linear models, a linear combination (addition of three policies) does not affect our analysis. It further reduces the number of parameters to estimate. Similarly, we combined Restrictions and Regulations of Businesses and Restriction and Regulation of Government Services. Health resources can also be used as a proxy for increased awareness among governments and citizens. So, we do not consider “Public Awareness Measures” announcements to avoid multicollinearity in the set of predictor variables.

Table D shows the correlation between different government policies implemented across countries. The correlation value between pairs of any two NPIs is not high (>0.7 as observed with community mobility across different locations in Google Community Mobility Reports), so we include all the following eight government policies in our model in Equation S8. We also include the Social Mobility in Parks and Transit Stations to check its correlation with NPIs. Changes (reduction during the early stages of the pandemic) in social mobility were induced by the introduction of NPIs. However, social mobility is a combination of institutional measures, e.g., NPIs, and individual measures, e.g., social mobility. The correlation between social mobility and any NPIs as shown in Table D is not high (>0.7) so we do not reject any further NPIs.

Table D. Correlation Between NPIs

|  | Health Resources | Restriction and Regulation of Businesses | Closure and Regulation of Schools | External Border Restrictions | Quarantine | Restrictions of Mass Gatherings | Social Distancing | Internal Border Restrictions | Mobility Parks | Mobility Transit Stations |
| --- | --- | --- | --- | --- | --- | --- | --- | --- | --- | --- |
| Health Resources | 1.00 | 0.32 | 0.04 | 0.56 | 0.34 | 0.26 | 0.35 | 0.49 | -0.26 | -0.26 |
| Restriction and Regulation of Businesses | 0.32 | 1.00 | 0.55 | 0.46 | 0.35 | 0.58 | 0.44 | 0.43 | -0.01 | -0.44 |
| Closure and Regulation of Schools | 0.04 | 0.55 | 1.00 | 0.16 | 0.25 | 0.52 | 0.15 | 0.40 | 0.02 | -0.40 |
| External Border Restrictions | 0.56 | 0.46 | 0.16 | 1.00 | 0.28 | 0.38 | 0.47 | 0.26 | -0.19 | -0.54 |
| Quarantine | 0.34 | 0.35 | 0.25 | 0.28 | 1.00 | 0.34 | 0.32 | 0.26 | 0.14 | 0.02 |
| Restrictions of Mass Gatherings | 0.26 | 0.58 | 0.52 | 0.38 | 0.34 | 1.00 | 0.35 | 0.37 | -0.01 | -0.38 |
| Social Distancing | 0.35 | 0.44 | 0.15 | 0.47 | 0.32 | 0.35 | 1.00 | 0.24 | -0.14 | -0.41 |
| Internal Border Restrictions | 0.49 | 0.43 | 0.40 | 0.26 | 0.26 | 0.37 | 0.24 | 1.00 | -0.21 | -0.26 |
| Mobility Parks | -0.26 | -0.01 | 0.02 | -0.19 | 0.14 | -0.01 | -0.14 | -0.21 | 1.00 | 0.51 |
| Mobility Transit Stations | -0.26 | -0.44 | -0.40 | -0.54 | 0.02 | -0.38 | -0.41 | -0.26 | 0.51 | 1.00 |

Fig K shows the policy implementation of each country for policies considered in this work (as described above). Fig L illustrates different countries that implemented each policy. Note that we normalize $s_{j,t,p}$ such that $s_{j,t,p}\in\left[ 0,1 \right] \forall j, \forall t, \forall p$.


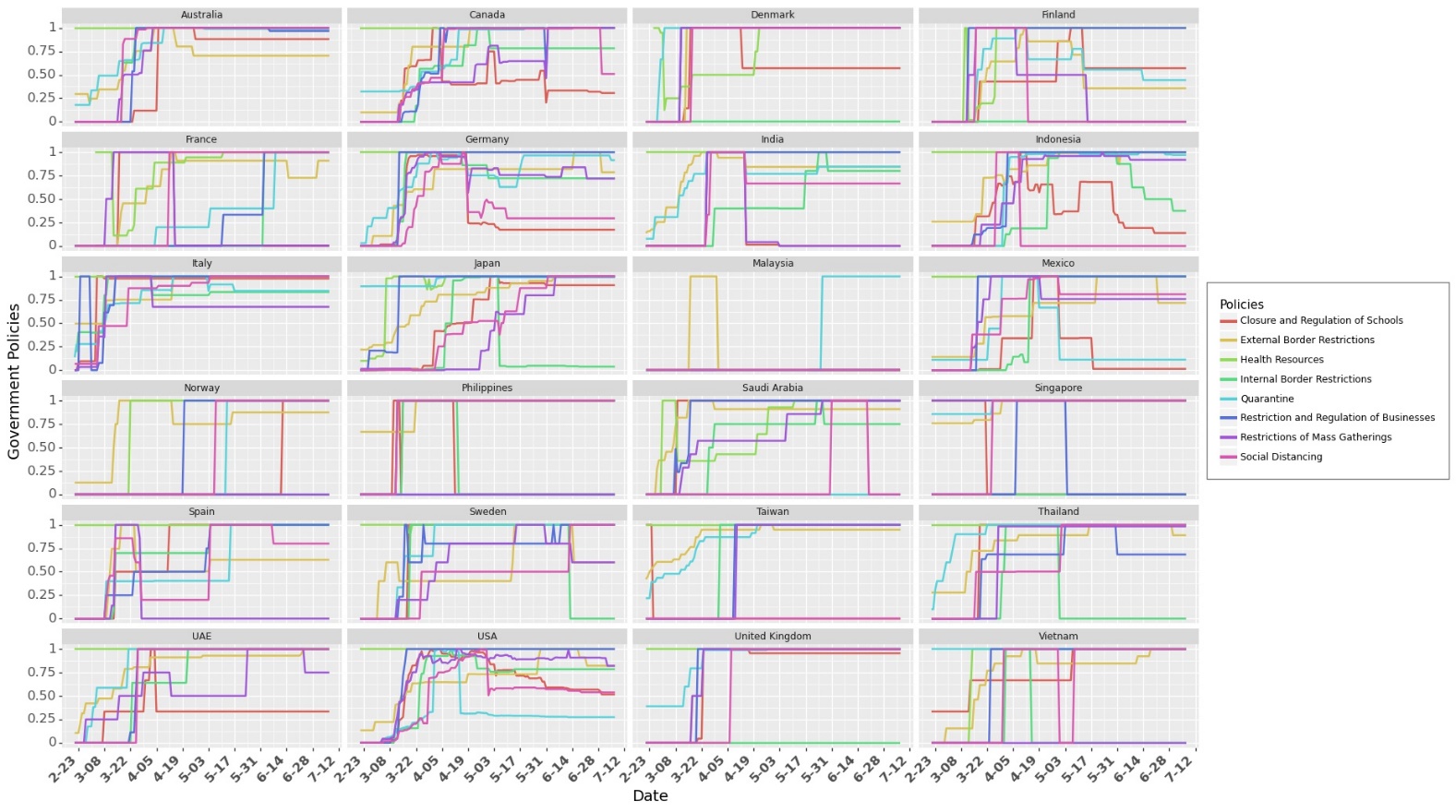


Fig K. Policy Implementation Across Different Countries. This graph shows how some countries, e.g., Taiwan and Malaysia employed only few of the policies. It also shows that countries, e.g., USA and Mexico employed region specific policies (at provincial level).


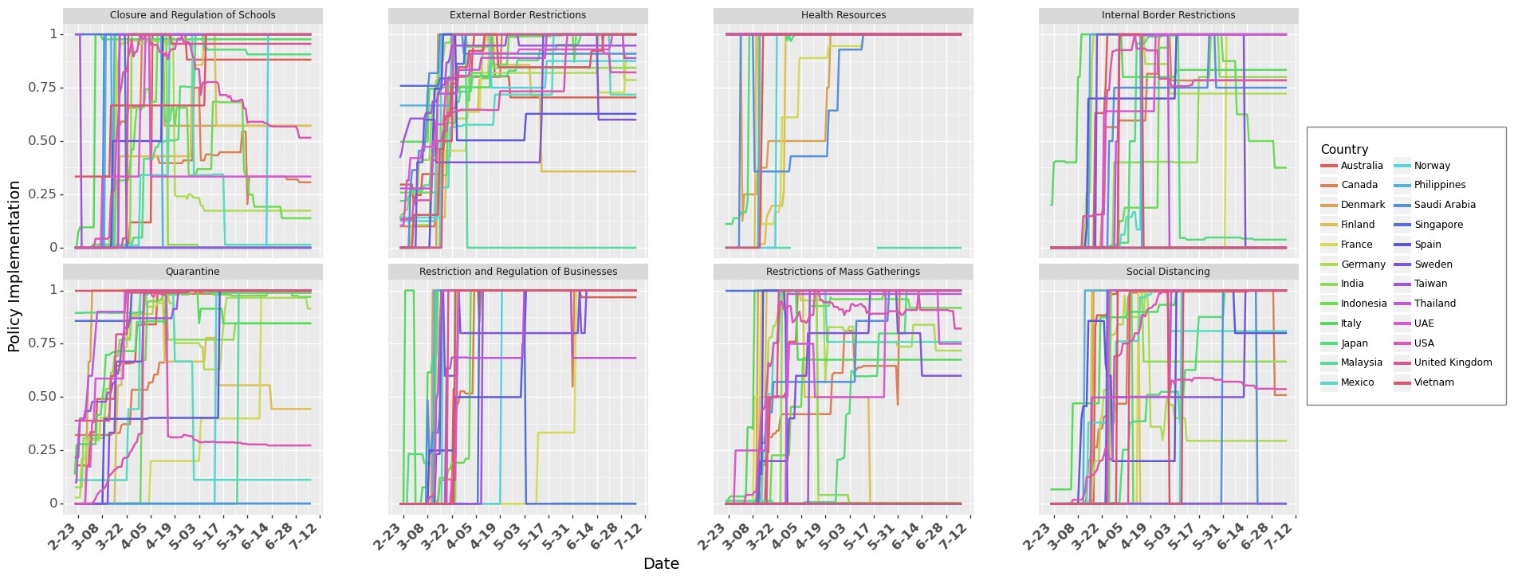


Fig L. Country wise Implementation of Different Policies. Fig shows how majority of the government policies were introduced at similar times across different countries. This makes it difficult to estimate the causal impact of each NPI on the spread of COVID-19.

**S2.6 Lag in Observation of the Effects of Control Variables**

Studies have reported a delay in observing the effects of policies on the events of a given day. This delay could be due to several reasons. One of the most prominent reasons is the incubation period (time between getting infected and onset of symptoms/knowing that individual is confirmed for COVID-19). During the incubation period, an individual may be asymptomatic. Incubation period is estimated to be 4 days to 14 days [2]. Another reason could be the testing time – the time it takes to get the confirmation of results. Due to limited healthcare professionals, there could be a long queue to get tested to get the results from testing centers.

To model this delay, we use a lag variable $shift$. We use the cross-validation method to find the lag with the best fit for the data. We test $shift\in[0,14]$ and observe that the model performs best at a *shift* of 9 days. We discuss this further in Section §Robustness check.

**S2.7 Testing**

Testing is critical in identifying infectious individuals. Once identified, these individuals can be quarantined or isolated from the public so that they do not spread to susceptible individuals. While people can get tested when they start showing symptoms, evidence reports that even asymptomatic individuals can spread the virus (50% of cases can be attributed to asymptomatic cases [13]) Since they do not show any symptoms, people around them (e.g., an asymptomatic young adult living with family) are less cautious and may get infected through them. It is critical to identify asymptomatic individuals as they can spread the virus unknowingly. This can be done by increased testing and contact tracing the individuals who have come in contact with positively tested for the COVID-19. Testing can be crucial in identifying COVID-19 positive individuals so that they can be quarantined (hospital or home isolation) or treated early when symptoms start showing.

As testing increases, the probability that more confirmed positive cases would be identified increases too. This will increase the empirical growth rate over time as more confirmed cases will be reported. This effect shows that a change in testing patterns over time could lead to bias (or underestimating the effect of NPIs and masks). We use testing data to account for increased testing over time to counter this time-sensitive bias. Fig M shows the data on total tests (per thousand people) in a country from ourworldindata.org [14]. In our analysis, we normalize the number for testing such that $0\leq testing_{j,t}\leq1 \forall j, \forall t$.


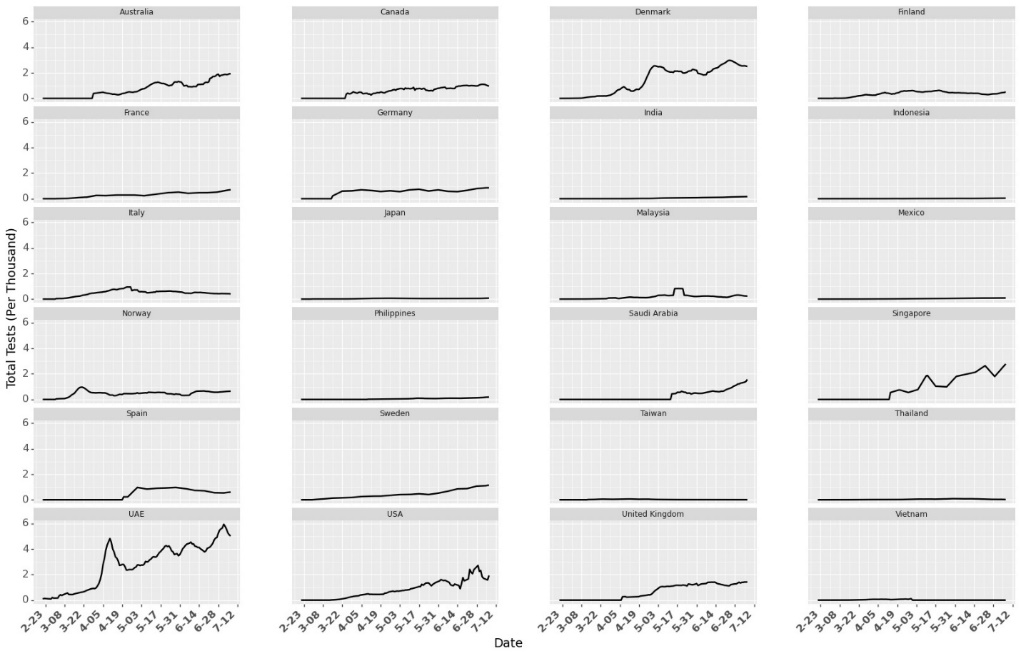


Fig M. Tests per Thousand in different countries over time. The numbers indicate the total number of tests conducted per thousand people in a country but the data set does not provide information on how many individuals were tested for COVID-19. Some individuals might get tested multiple times.

**S2.8 Google Trends**

As the number of cases increases, awareness increases in public (e.g., washing hands more often). We use Google Trends [15] to account for the increase in active awareness over time (Fig N). Google Trends numbers indicate the search interest of a topic over time as a proportion of all other searches at the same time. In our analysis, we normalize the number for Google Trends such that $0\leq trend_{j,t}\leq1 \forall j, \forall t$.


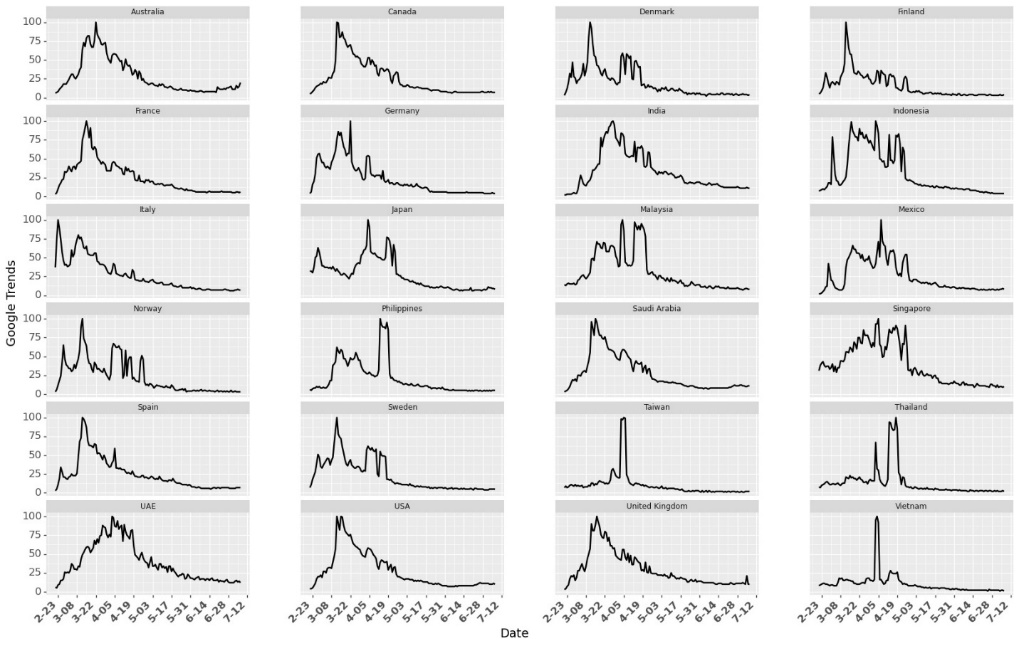


Fig N. Google Trends for the Search term *coronavirus* in different countries.

**S2.9 Week Fixed Effects**

COVID-19 containment changes over time. It includes increasing public awareness or better understanding of the virus as more studies and research come to public attention. Not only do citizens understand how to be more careful (or more informed), healthcare providers also learn more about the disease for more efficient treatment of COVID-19 patients (e.g., creating new wards for COVID-19 patients, treating them by wearing Personal Protection Kits, PPE). It also involves improved infrastructure, e.g., testing or converting existing medical facilities to dedicated COVID-19 centers. To account for all the time-sensitive fixed effects (other than the controls we discussed before), we use fixed effects for weeks (from the day that country reaches $th$ in our analysis).

Fig O shows how the growth rate changes across different weeks (Fig P provides the same information across different countries). Even after removing the initial noisy data, we observe the highest variance in the growth rates during week 1 in most countries (Fig O and Fig P). We use one-hot vector to denote week (${week}_{j,t,w}=1)$ if day $t$ lies in week $t$ for country $j$. Note that due to different starting times for each country (Fig F), a day may come under a different week for a different country. For example, days in week 1 for Vietnam are earlier in the calendar than the days in week 1 in India. Note that the growth rate is higher during the initial weeks and slows down with time. To capture this effect, we use fixed effects for weeks. Results in Section §Result show that the magnitude of coefficient for week 0 is higher than the magnitude of the coefficient for week one and so on.


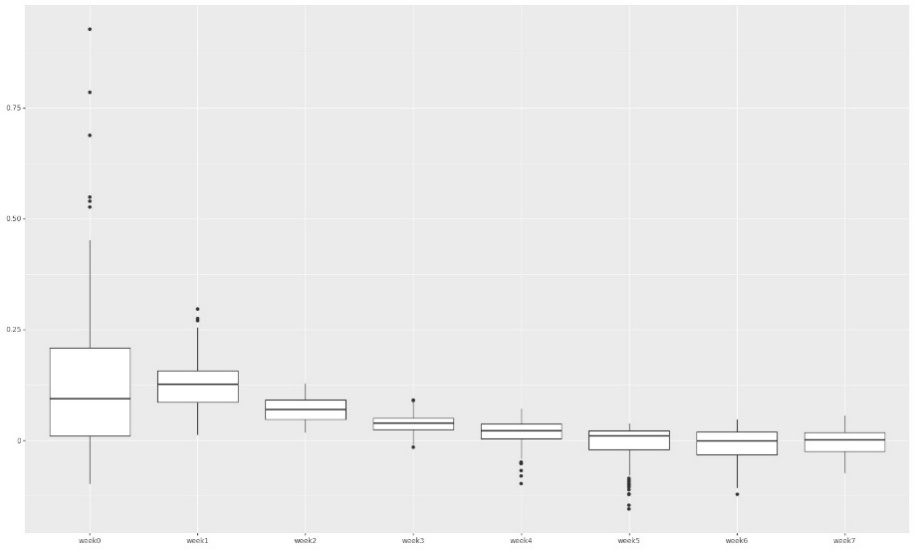


Fig O. Box plot for growth rate of different countries across different weeks.


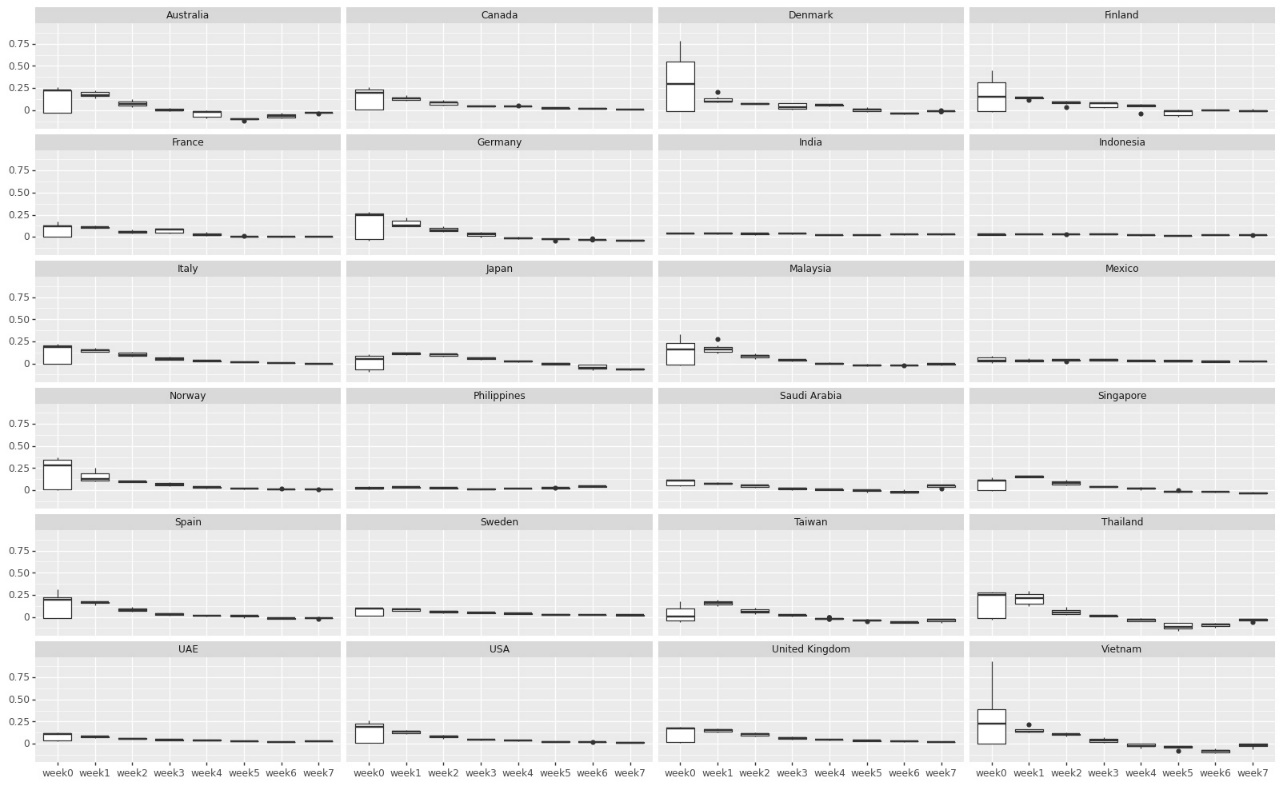


Fig P. Box plot for growth rate in different weeks across different countries.

**S2.10 Country Fixed Effects**

We observe heterogeneity across countries with many aspects in handling COVID-19. Multiple factors affect the spread and handling of a disease in a country. Heterogeneity may be observed at different levels. For example, heterogeneity at the government level includes the difference in reporting cases, testing infrastructure, strictness is reducing social mobility, and implementation of NPIs. Population-wise heterogeneity includes population density in a country or percentage of the population living in high-density urban regions or poor neighborhoods with shared sanitation facilities. It may also include cities with international airports or travelers (particularly from countries hard hit with COVID-19 in early 2020, e.g., China and Iran). It also includes heterogeneity at the level of education (awareness about COVID-19, responsibility in understanding the severity of precautions), poverty (health insurance, ability to purchase sanitizers or high-quality masks), primary health care facilities (drinking water, sanitation, shared places) or family structure (number of young adults in a family or size of the family residing in a residential complex). To control for all this heterogeneity among countries which may lead to country-level effects in the growth rate of COVD-19, we use country fixed effects.

Note that since we use a constant in our model, we consider fixed effects for 23 countries, and we consider the last country (Vietnam) as our base country (with 0 fixed country effect). This ensures that parameter estimates are stable.

**S3. Additional Results**

We use the growth rate model to study the effect of Masks, Social Mobility, and Non-Pharmaceutical Interventions (NPIs). Since we do not have real numbers on how many people wear masks (or wear masks that could be effective), we use different transformations of the mask numbers from the surveys. We use growth rate model with masks transformed as $ln(1+mask_{j,t})$ as our focal model. We use threshold $th=0.2$ and $shift=9$ days. Details on selection of $th$ and $shift$ is provided in Section §Robustness Check. Model statistics are given in Table E. Table F shows the parameter estimates for the model with different transformations.

Table E. Model statistics

| R-squared: | 0.738 |
| --- | --- |
| Adj. R-squared: | 0.729 |
| F-statistic: | 88.03 |
| Prob (F-statistic): | 0 |
| Log-Likelihood: | 2400.8 |
| AIC: | -4712 |
| BIC: | -4475 |
| No. Observations: | 1422 |
| Df Residuals: | 1377 |
| Df Model: | 44 |

Table F. Parameter Estimates for Growth Rate Model

| var | coefficient | std error | t-value | p-value | lower limit | upper limit |
| --- | --- | --- | --- | --- | --- | --- |
| const | 0.1999 | 0.017 | 11.745 | 0 | 0.167 | 0.233 |
| log(Mask) | -0.1047 | 0.024 | -4.385 | 0 | -0.152 | -0.058 |
| Mobility Parks | -0.0296 | 0.006 | -4.677 | 0 | -0.042 | -0.017 |
| Mobility Transit Stations | 0.1109 | 0.013 | 8.398 | 0 | 0.085 | 0.137 |
| week0 | 0.0981 | 0.009 | 11.025 | 0 | 0.081 | 0.116 |
| week1 | 0.0589 | 0.009 | 6.872 | 0 | 0.042 | 0.076 |
| week2 | 0.0411 | 0.008 | 5.146 | 0 | 0.025 | 0.057 |
| week3 | 0.0324 | 0.007 | 4.335 | 0 | 0.018 | 0.047 |
| week4 | 0.018 | 0.007 | 2.575 | 0.01 | 0.004 | 0.032 |
| week5 | 0.0039 | 0.007 | 0.592 | 0.554 | -0.009 | 0.017 |
| week6 | -0.0013 | 0.006 | -0.209 | 0.834 | -0.014 | 0.011 |
| week7 | 0.0021 | 0.006 | 0.343 | 0.732 | -0.01 | 0.014 |
| Testing | -0.0121 | 0.006 | -1.938 | 0.053 | -0.024 | 0 |
| Trend | -0.0455 | 0.008 | -5.933 | 0 | -0.061 | -0.03 |
| Health Resources | -0.034 | 0.012 | -2.881 | 0.004 | -0.057 | -0.011 |
| Restriction and Regulation of Businesses | -0.0049 | 0.005 | -1.03 | 0.303 | -0.014 | 0.004 |
| Closure and Regulation of Schools | -0.0153 | 0.006 | -2.436 | 0.015 | -0.028 | -0.003 |
| External Border Restrictions | -0.0315 | 0.008 | -3.807 | 0 | -0.048 | -0.015 |
| Quarantine | -0.0321 | 0.01 | -3.194 | 0.001 | -0.052 | -0.012 |
| Restrictions of Mass Gatherings | -0.0066 | 0.007 | -1.007 | 0.314 | -0.019 | 0.006 |
| Social Distancing | 0.0038 | 0.006 | 0.618 | 0.536 | -0.008 | 0.016 |
| Internal Border Restrictions | -0.01 | 0.006 | -1.639 | 0.101 | -0.022 | 0.002 |
| Australia | -0.0338 | 0.013 | -2.531 | 0.011 | -0.06 | -0.008 |
| Canada | 0.0205 | 0.012 | 1.645 | 0.1 | -0.004 | 0.045 |
| Denmark | 0.0167 | 0.014 | 1.23 | 0.219 | -0.01 | 0.043 |
| Finland | -0.02 | 0.016 | -1.27 | 0.204 | -0.051 | 0.011 |
| France | -0.032 | 0.014 | -2.209 | 0.027 | -0.06 | -0.004 |
| Germany | -0.0101 | 0.014 | -0.739 | 0.46 | -0.037 | 0.017 |
| India | -0.0038 | 0.013 | -0.295 | 0.768 | -0.029 | 0.022 |
| Indonesia | 0.0353 | 0.013 | 2.71 | 0.007 | 0.01 | 0.061 |
| Italy | 0.0481 | 0.011 | 4.308 | 0 | 0.026 | 0.07 |
| Japan | -0.0034 | 0.01 | -0.348 | 0.728 | -0.022 | 0.016 |
| Malaysia | -0.0397 | 0.013 | -2.944 | 0.003 | -0.066 | -0.013 |
| Mexico | 0.0002 | 0.017 | 0.014 | 0.989 | -0.032 | 0.033 |
| Norway | -0.0373 | 0.016 | -2.284 | 0.023 | -0.069 | -0.005 |
| Philippines | -0.0315 | 0.017 | -1.88 | 0.06 | -0.064 | 0.001 |
| UAE | 0.1119 | 0.025 | 4.418 | 0 | 0.062 | 0.162 |
| Saudi Arabia | 0.015 | 0.016 | 0.949 | 0.343 | -0.016 | 0.046 |
| Singapore | 0.0461 | 0.015 | 3.124 | 0.002 | 0.017 | 0.075 |
| Spain | 0.0073 | 0.014 | 0.543 | 0.588 | -0.019 | 0.034 |
| Sweden | -0.0248 | 0.014 | -1.776 | 0.076 | -0.052 | 0.003 |
| Taiwan | -0.0294 | 0.013 | -2.322 | 0.02 | -0.054 | -0.005 |
| Thailand | 0.0209 | 0.012 | 1.81 | 0.071 | -0.002 | 0.044 |
| United Kingdom | 0.0215 | 0.014 | 1.547 | 0.122 | -0.006 | 0.049 |
| USA | 0.0252 | 0.013 | 1.872 | 0.061 | -0.001 | 0.052 |

Fig Q shows the complete results for the parameter estimation under different transformations for masks. Results for parameter estimation in Table F show consistency in the estimates for social mobility and NPIs. They also show consistent parameter estimates for fixed effects (week and countries). Fixed effect of week can capture the trend in awareness or infrastructure change over time. At the beginning of the pandemic in a country, the growth rates were higher. This higher growth rate corroborates positive and statistically significant values for the fixed effects of weeks (decreases as weeks increase). The coefficients for masks seem different across the different transformations, but due to their transformation, it has to be interpreted differently (as we discuss next in the interpretation of results). However, we cannot claim causality from these results as the NPIs were not randomly introduced in different countries. Nonetheless, we can estimate the combined effect of different mobilities and NPIs.


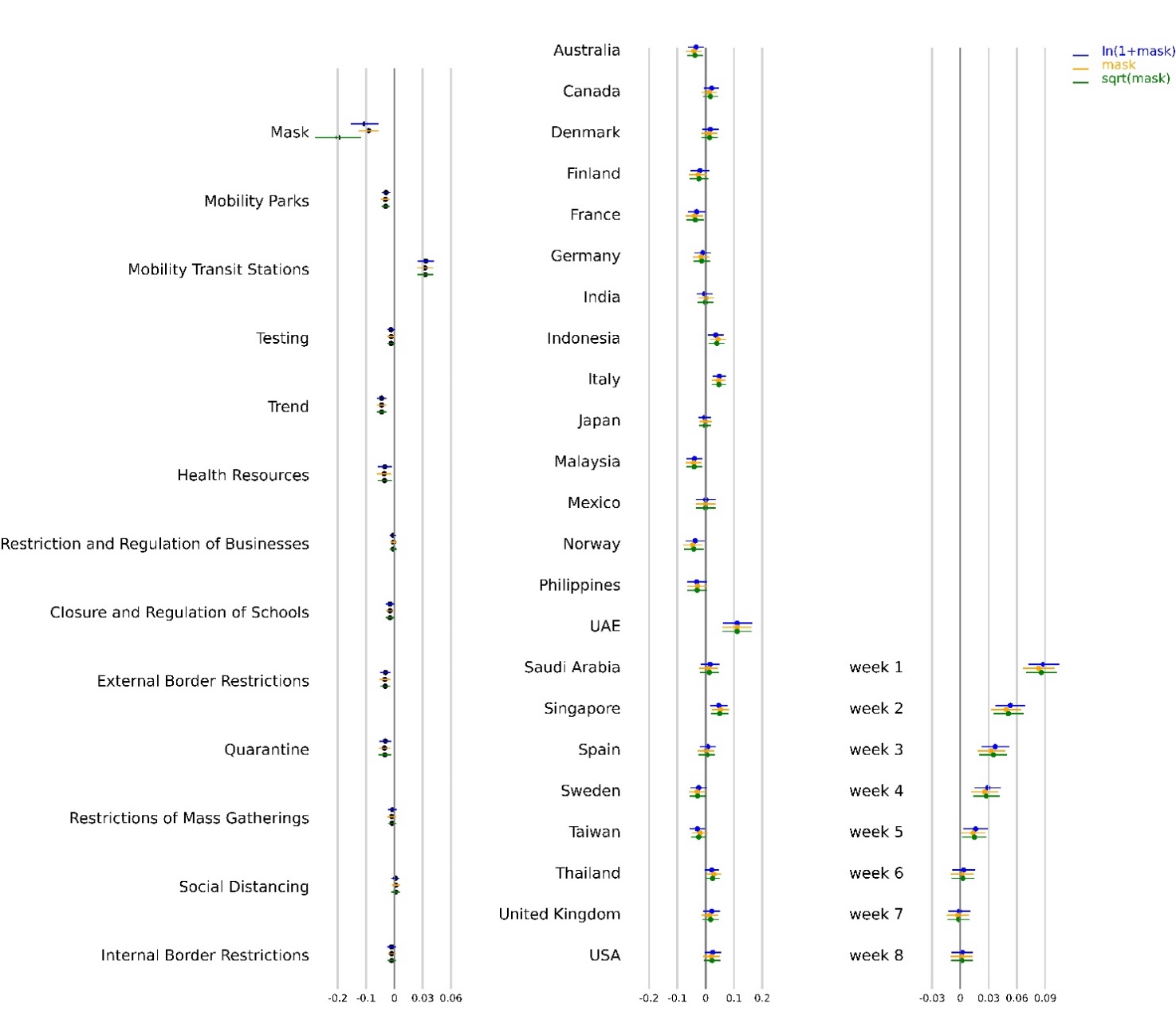


Fig Q. Parameter estimates for models with different transformations for masks. We use $th=0.2$ and $shift$= 9 days.

**S3.1 Krinsky-Robb method**

In the rest of our analysis, we use the Krinsky-Robb method to estimate confidence intervals for the combined effect of masks, social mobility, and NPIs. We also use this method to obtain confidence bounds across the growth rate predictions and active cases in a country. Krinsky-Robb method is a Monte Carlo simulation method used to draw samples from a multivariate normal distribution. We use the ordinary least square method to estimate the coefficients $\theta$ in Equation S8. The ordinary least square method for multiple linear regression assumes a multivariate normal distribution of $\theta$. Krinsky-Robb method takes advantage of this assumption to sample random draws for $\theta$ using Cholesky decomposition and standard normal variates. Steps in the Krinsky-Robb method are:

1. Find Cholesky decomposition matrix $C$ for the covariance matrix of $\sum_{\theta} .$
2. Draw $\left| \theta\right| \times n$random samples, $x_{samples}$, from standard normal distribution ($|\theta|$ is the cardinality of $\theta$).
3. $\theta_{samples}=\hat{\theta}+C \times x_{samples}$($\hat{\theta}$ is the estimated coefficient or variable).
4. Calculate confidence interval based on $\theta_{samples}$.

We use this method to get confidence interval bounds for the sum of the coefficients of mobility and NPIs to get the combined effect. We also use this method to predict confidence intervals of growth rate and daily active cases under different scenarios, as we discuss next. First, we discuss the model performance and then discuss the interpretation of the coefficients in Table F.

**S3.2 Model Performance**

We can use the coefficients from our model to predict the growth rate for different countries. Fig R shows the actual growth rate (green dots) with the predicted growth rate (blue line) and its confidence interval (blue shade). We use the Krinsky-Robb method to estimate the confidence interval bounds around the prediction. Results show that the model can accurately predict the growth rate of daily infections across different countries. Green and Brown vertical lines indicate the 60 days period for which data was collected for that country.


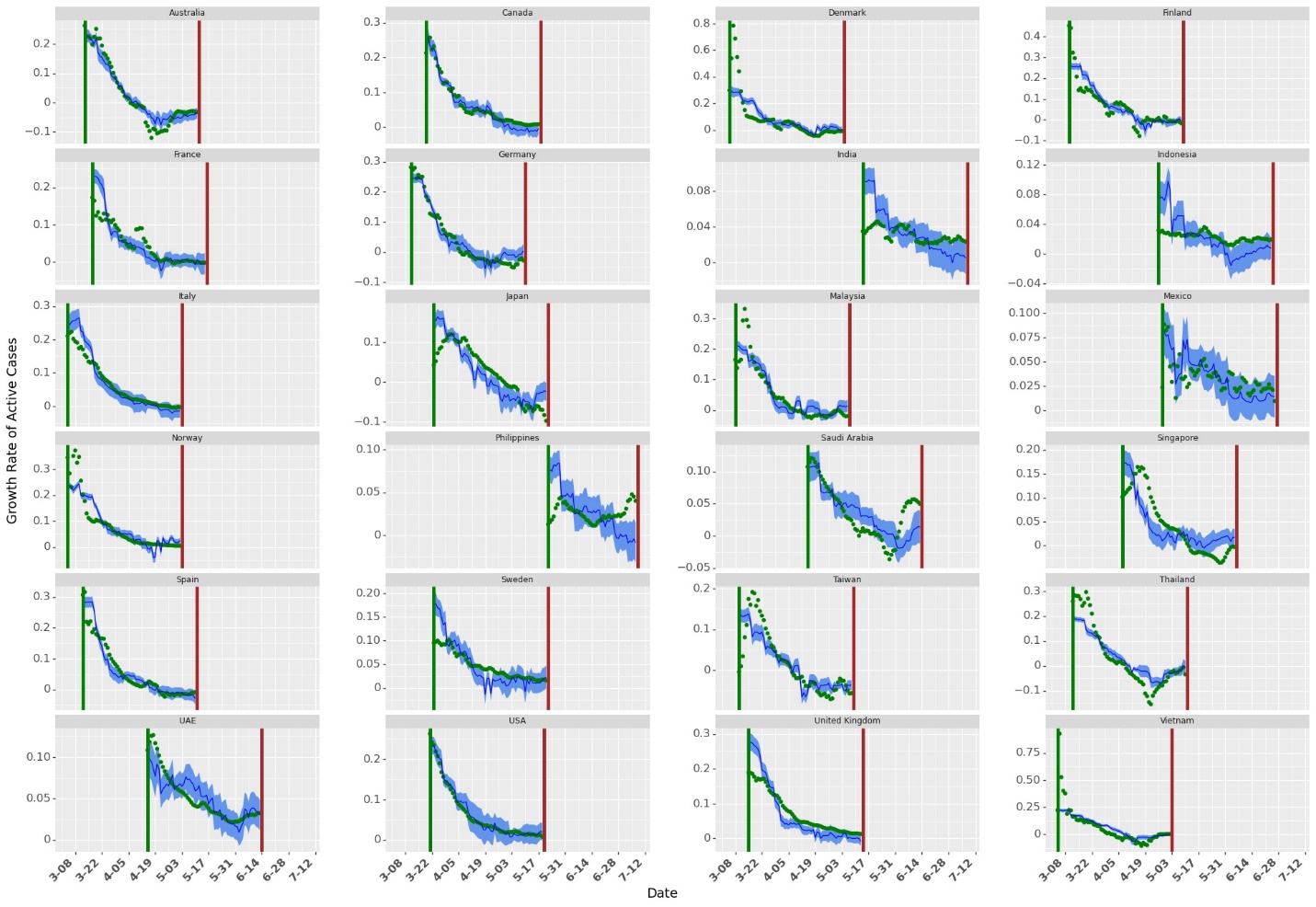


Fig R. Growth Rate Predictions for different countries using current data. The green dots show the actual growth rates across countries. The dark blue line shows the mean of growth rate prediction (for 10000 samples in the Krinksky-Robb method). The blue shaded area shows the confidence bounds around the mean prediction.

Since growth rate is a forward-looking model, we can also use growth rates to estimate active infectious population by $I_{j,t}=I_{j,t-1}\times g_{j,t}$. Fig S shows the results for daily active cases. Note that we estimate active cases using an exponential model. Thus, as the number of days in the prediction model increases, confidence interval bounds around predictions increase. However, the mean prediction for active cases closely approximates the actual active cases for different countries.


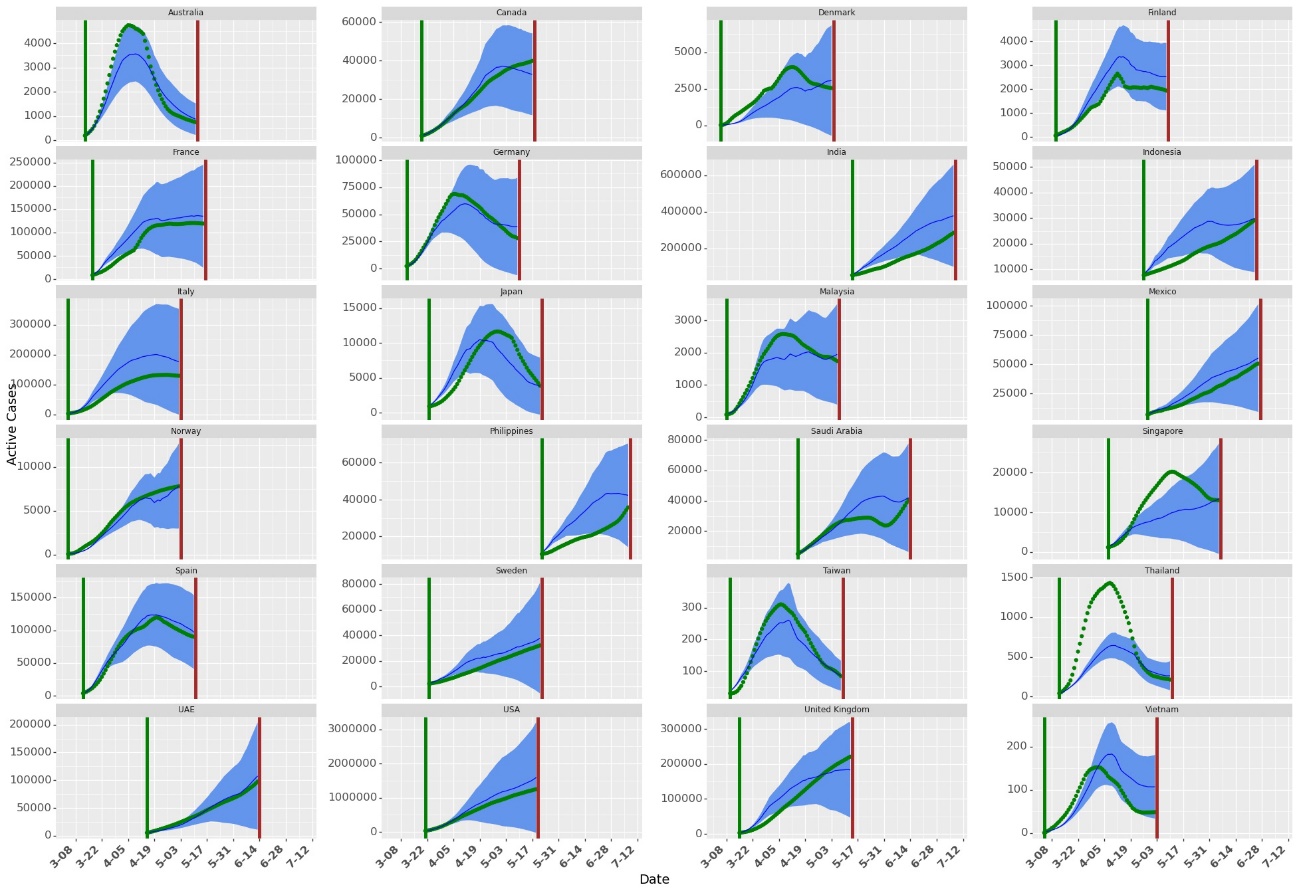


Fig S. Simulating Daily Active Cases using Forward-Looking Growth Rate Model. The green dots show the actual daily active cases across countries. The dark blue line shows the mean of daily active cases prediction (for 10000 samples in the Krinksky-Robb method). The blue shaded area shows the confidence bounds around the mean prediction. As the days increase, the confidence bounds increase due to the multiplicative nature of forward-looking growth rate models.

**S3.3 Effect of Masks, Social Mobility and NPIs**

We model growth rate as the first difference of log of daily active confirmed infectious cases as shown in Equation S7. Thus, the exponential of the coefficients in Table F (other than the mask as we discuss next) estimates % drop in active cases on the day $t$(as compared to active cases on the day $t-1$). Using the coefficients in Table F, we can estimate the combined effect of masks, social mobility, and NPIs by using the Krinsky-Robb method.

**S3.3.1 Mask**

Negative and statistically significant coefficient for masks show that increased mask wearing behavior may lead to a decrease in the growth rate of COVID-19. As we use different transformations, we should interpret the coefficient for masks differently, too. When masks are transformed as $ln(1+mask)$, a coefficient of $\theta_{m}$ shows that if 100% of the population wears masks, it would lead to a daily drop of ${1-e}^{\theta_{m}(\ln\left( 1+1 \right)-\ln\left( 1+0 \right))}$ % in the growth rate as compared with the scenario when no one wears face masks. For raw mask numbers, the effect of masks can be interpreted directly as $1-e^{\theta_{m}} \%$ drop in daily total infectious cases when everyone wears masks as compared to no one wearing masks. When masks are transformed as $\surd\left( 1+mask \right),$a coefficient of $\theta_{m}$ shows that if 100% of the population wears masks, it would lead to a daily drop of ${1-e}^{\theta_{m}(\surd\left( 1+1 \right)-\surd\left( 1+0 \right))}$ % in the growth rate as compared with the scenario when no one wears face mask. Similarly, we can estimate the bounds for the effect of coefficients of masks. Table G provides the estimate for the decrease in daily growth rate when 100% additional population wears face masks in public spaces (under different transformations).

Table G. Daily Drop in the Growth Rate When Additional 100% People Wear Face Masks

| **Masks** | **Lower Limit of Daily Drop** | **Daily Drop** | **Upper Limit of Daily Drop** |
| --- | --- | --- | --- |
| $ln(1+mask)$ | 3.8% | 6.9% | 10.1% |
| $mask$ | 5.7% | 9.1% | 12.5% |
| $\surd(1+mask)$ | 4.9% | 8.2% | 11.4% |

Fig T illustrates the effects of not wearing masks in each country. The results are not significantly different for Denmark, Finland, Norway, and Sweden as these countries already had very low numbers for mass wearing in public spaces. Similarly, the effect is much more substantial for countries, e.g., Japan, Thailand, and Vietnam, with higher percentages of people wearing face masks.


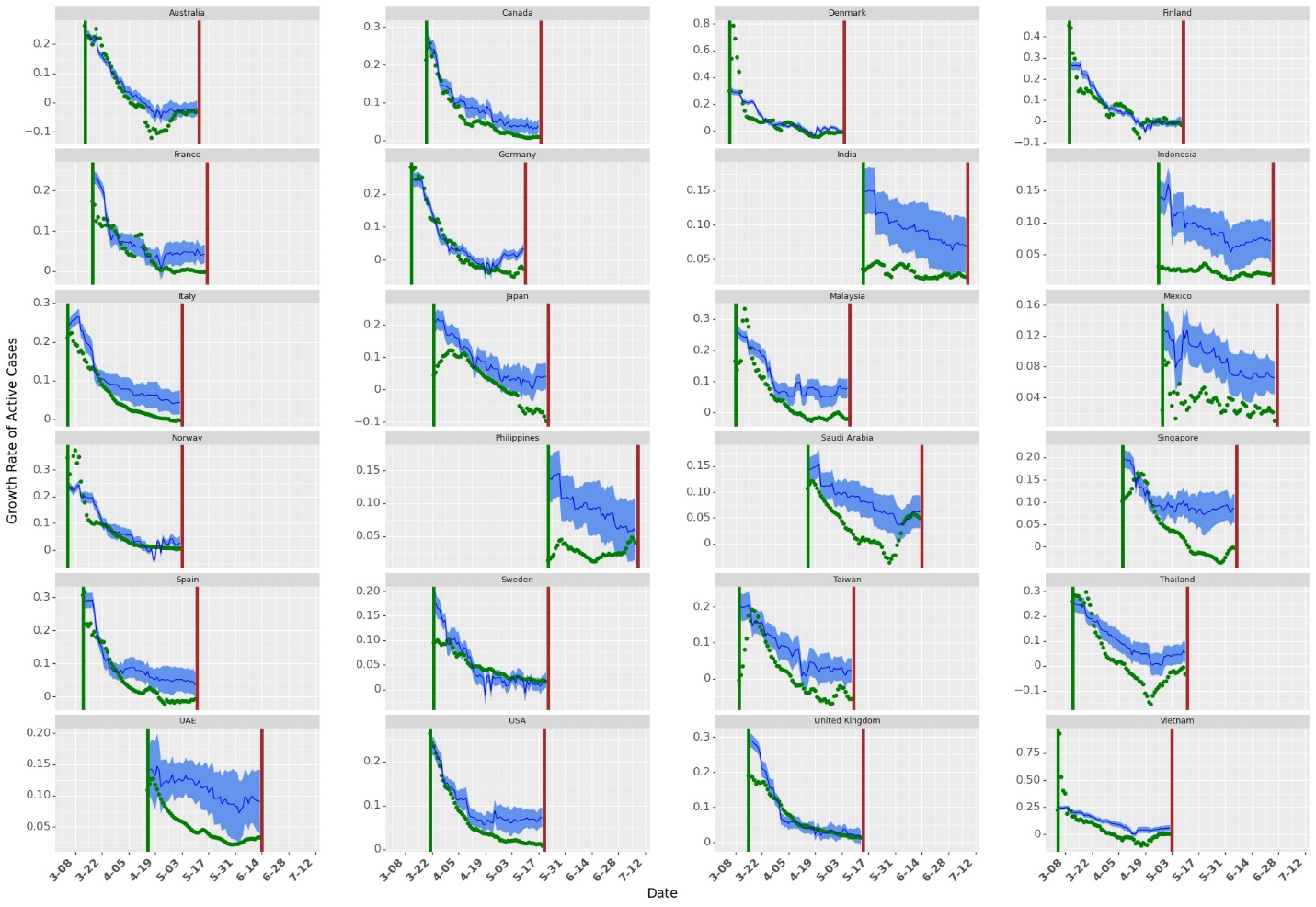


Fig T. Growth Rate with no Mask Wearing. The green dots show the actual growth rates across countries. The dark blue line shows the mean of growth rate prediction (for 10000 samples in the Krinksky-Robb method). The blue shaded area shows the confidence bounds around the mean prediction. We transform the masks to $ln(1+mask)$ to predict the growth rate.

Similar to Fig S, we can predict daily active cases with zero percent mask wearing as shown in Fig U. The results show that masks lead to a significant reduction in total cases as without these measures, the number of cases could exponentially increase over time (more discussion later on §Country-wise effect).


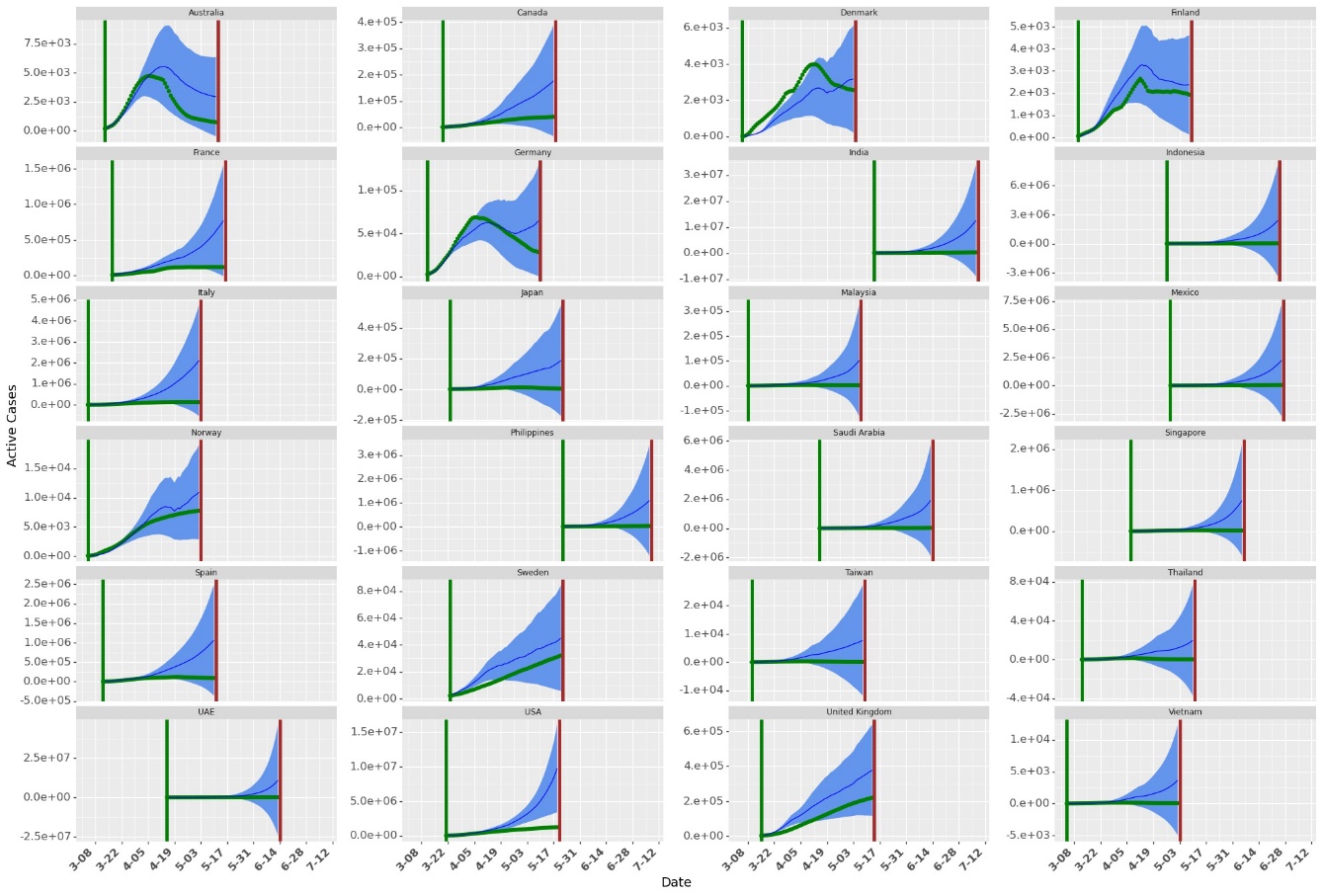


Fig U. Simulation with no Mask Wearing. The green dots show the actual growth rates across countries. The dark blue line shows the mean of active cases prediction (for 10000 samples in the Krinksky-Robb method). The blue shaded area shows the confidence bounds around the mean prediction.

We build five simulation models to further understand the impact of masks. In the first simulation model, we consider a hypothetical country with a constant value for all the covariates in Equation S8. In the second simulation model, we check the change in active cases at the end of 60 days when mask wearing in a country is $m\%$ where $m\in[0,10,20,..,100]$. In the third model, we check the change in active cases at the end of 60 days if the current levels of mask wearing are multiplied by a factor of $x\in\left[ 0,0.2,0.4,\ldots,2 \right].$ In the fourth model, we present results for active cases at the end of 60 days when mask wearing percentage increases by $a\%$ compared to the current levels in that country where $a\in[1,2,\ldots,10]$. In the fifth model, we exchange the mask wearing numbers between countries with minimum and maximum average mask wearing through the period of our analysis.

Simulation Model 1 helps isolate the effect of masks in this analysis; we do not consider any other covariates (as if no individual or institutional measures were taken apart from wearing masks in public areas). We construct data for a hypothetical country with these numbers to quantify the effect of masks in our analysis. In Simulation Model 2-4, we study the country-wise association of masks with growth rate. In these models, we do not change the numbers of any other covariates other than masks. These results show the potential change in active cases in that country for different percentages of people wearing masks in public. Simulation model 5 is used to build an approximate counter factual model for masks by exchanging mask wearing in countries with minimum and maximum average mask wearing during our analysis. We discuss the results of these simulation models next.

*Simulation Model 1: Average country*

We simulate a hypothetical country with no mask wearing ($mask_{j,t}=0),$ no active awareness $\left( trend_{j,t}=0 \right),$ no testing$\left( testing_{j,t}=0 \right),$ and no government implemented NPIs ($s_{j,t,p}=0)$. We predict the active cases at the end of 60 days at different levels of mask wearing. We consider the average country with 0 country fixed effects for prediction. Fig V shows the daily active cases for this average country at different levels of mask wearing. We assume that the average country has 100 cases on day 0 in the simulation. Results show that increasing the mask number can help flatten the curve (even when social mobility and NPIs remain unchanged). As the percentage of people wearing face masks increases, the daily active cases go down compared to no mask wearing. Results also show that social mobility and NPI can play a significant role in flattening the curve (the daily active curve flatten even with no mask wearing, albeit slower). Cases start rising again after initial flattening for most cases as social mobility increases and NPIs are relaxed (Fig E andFig F). The results imply that if masks are mandated and become widespread, complete lockdowns may be eased to help alleviate the associated economic hardships.


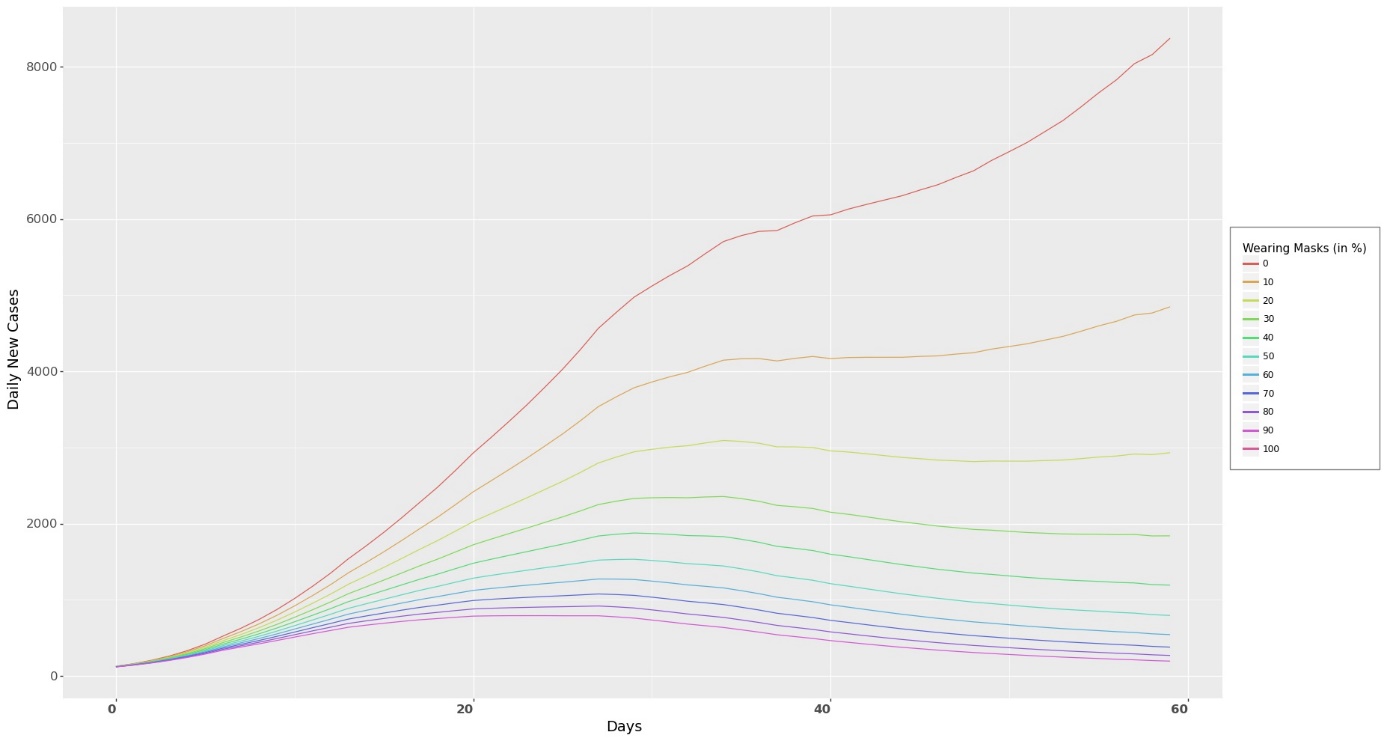


Daily Active Cases

Fig V. Daily Active Cases for the Average Country. As more people wear masks, the faster the curve can be flattened. Note that the graph shows the mean prediction under different levels of mask wearing. Also, it shows the daily active cases. Flattening of a curve has been synonymous with daily new cases, but if we use $I_{t}-I_{t-1}$, we can approximate daily new cases.

*Simulation Model 2: Changing mask levels*

In this simulation, we predict the number of active cases in each country by changing the levels of mask wearing. Fig W shows the ratio of active cases at the end of 60 days under different levels of mask wearing as compared to active cases at the current levels of masks. As the mask levels increase, the ratio of active cases to the actual active cases at the end of 60 days decreases.


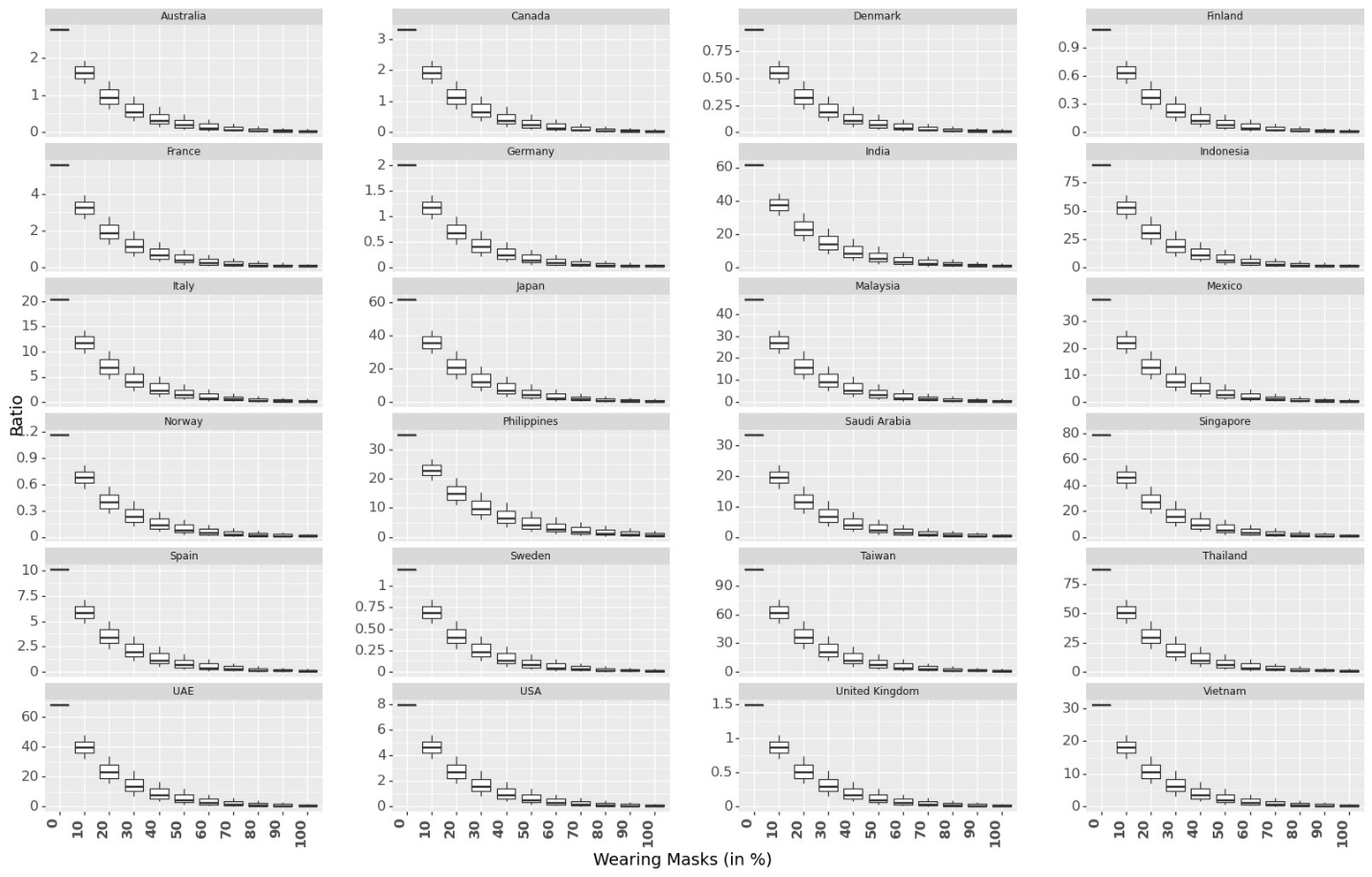


Fig W. Box Plot on Ratio of Predicted Active Cases to Actual Cases at the End of 60 Days Under Different Levels of Mask Wearing.

*Simulation Model 3: Multiplying a constant to current mask levels*

In this simulation, we multiply the current mask wearing levels with a constant multiplication factor ($\in$[0.2, 0.4, …, 2]) to predict active cases at the end of 60 days as compared to actual cases across 24 countries (Fig X). Similar to Fig W, we observe that as we increase mask levels, the ratio decreases significantly. However, the effect is different across different countries.

Similar to results in Fig W, when the mask levels are much lower than the current levels (e.g., countries like Thailand, Vietnam, Singapore), the ratio of active cases at the end of 60 days to their actual cases is much higher compared to countries with lower current mask rates (e.g., Sweden, Norway).


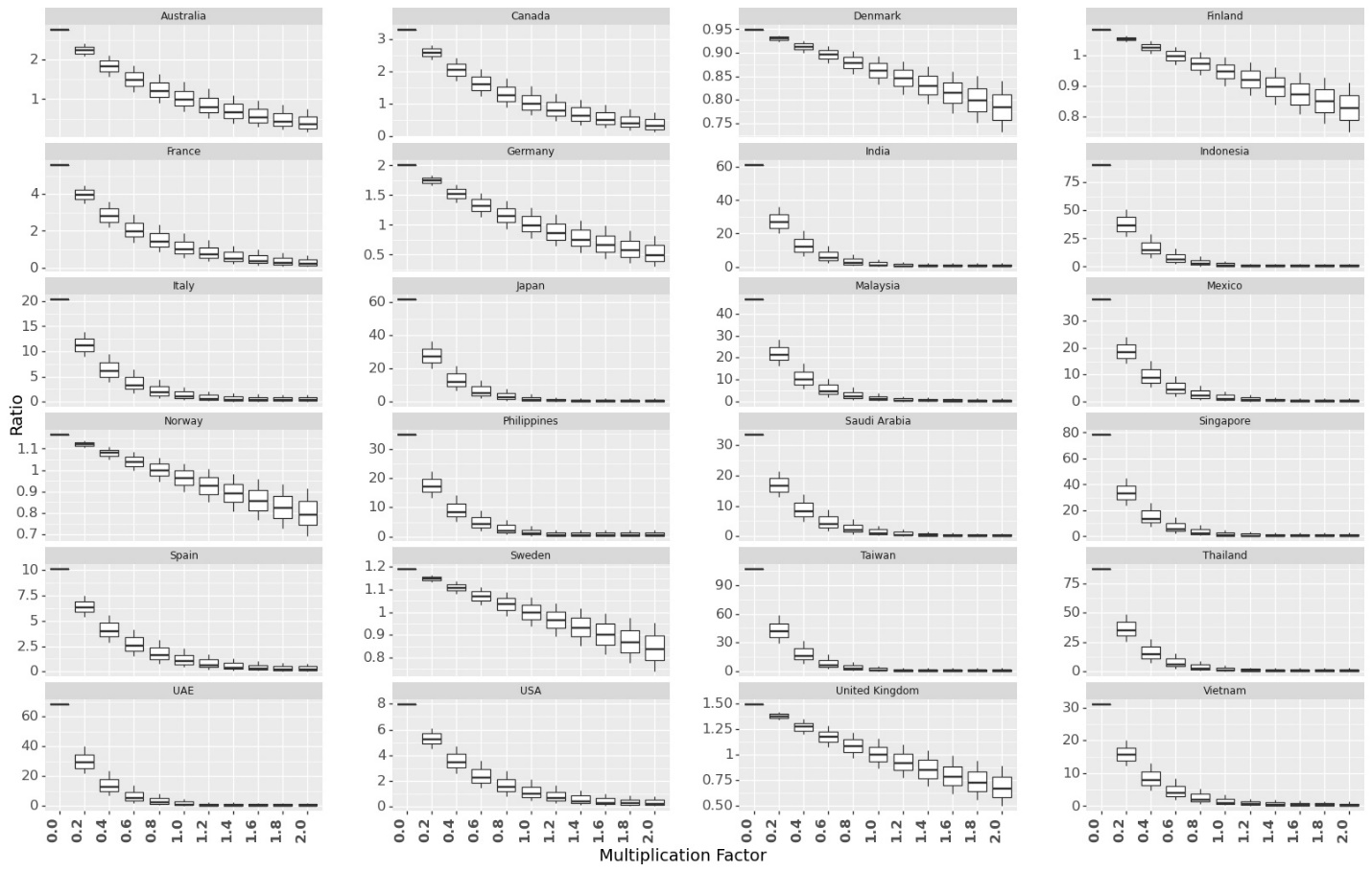


Fig X. Box Plot on Ratio of Predicted Active Cases to Actual Cases at the End of 60 Days Under Different Levels of Mask Wearing obtained by multiplying the current levels of mask wearing with a constant.

*Simulation Model 4: Adding a constant to current mask levels*

In this simulation, we predict the ratio of active cases at the end of 60 days when mask wearing in a country is increased by different percentage points (0%, 1%, 2%, …, 9%). Fig Y plots the ratio of active cases at the end of 60 days with simulation for increased mask wearing to the actual active cases. This could help the government form policies that if $a$% of more people follow the mask wearing guidelines, which NPIs they could relax while still controlling the spread of the virus. Enforcing a mask wearing policy could be particularly useful in countries with low mask wearing.


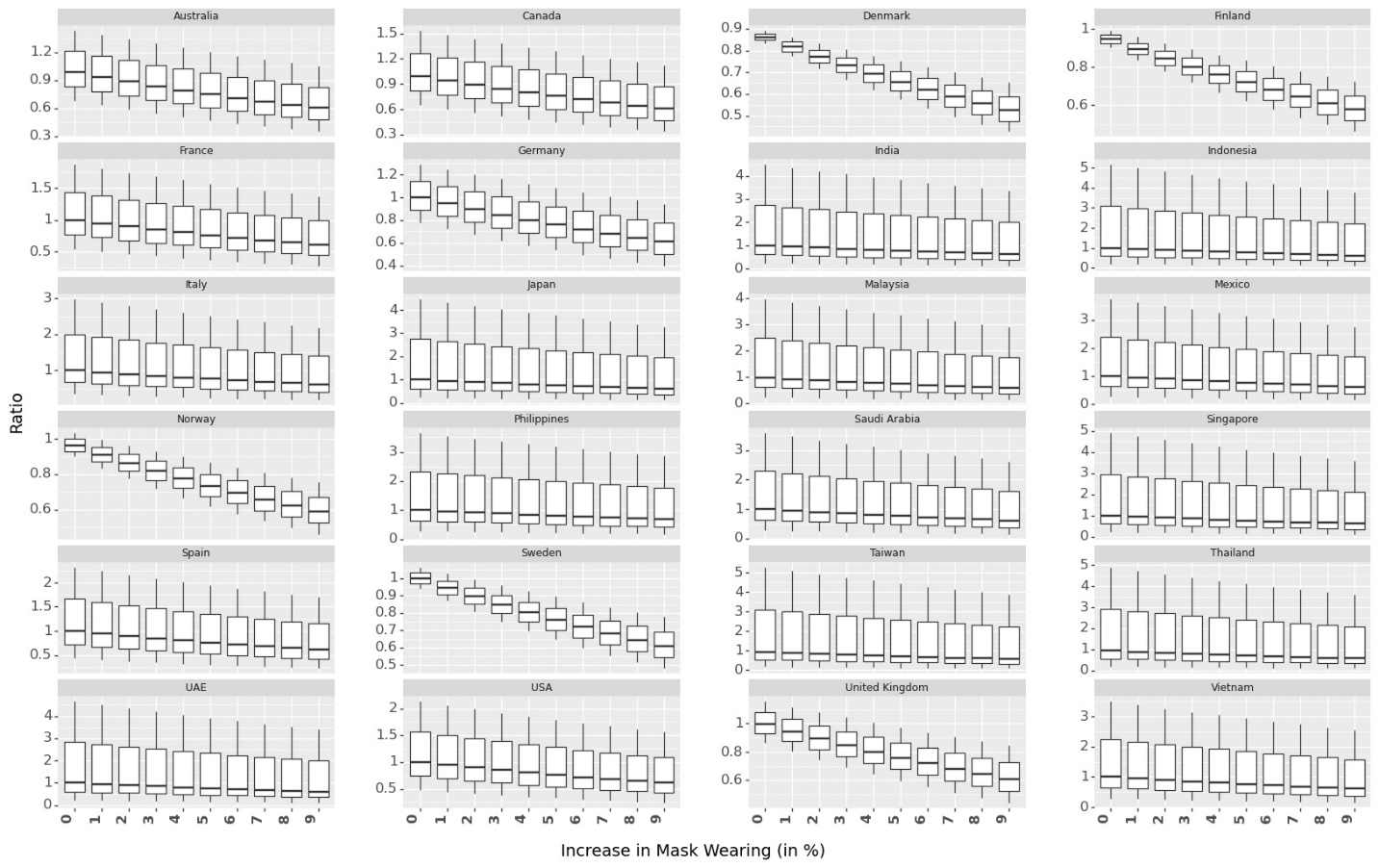


Fig Y. Box Plot on Ratio of Predicted Active Cases to Actual Cases at the End of 60 Days Under Different Levels of Mask Wearing obtained by increasing the current levels of mask wearing by different percentage points.

*Simulation Model 5: Exchanging the current mask levels*

Countries have observable heterogeneity in their culture of mask wearing (Fig D). Mask wearing has been more common in Asian countries than in Scandinavian countries. In this simulation, we exchange the mask wearing numbers between 8 countries (4 Asian with highest average mask wearing among 24 countries and 4 Scandinavian countries with lowest average mask wearing among 24 countries). The ratio of active cases under new mask wearing as compared to actual active cases at the end of 60 days is shown in Table H. Results show that Scandinavian countries could have reduced their confirmed cases significantly if they had enforced people to wear face masks in public. We find that the Scandinavian countries could have reduced the active cases by up to 50 times in 60 days if the citizens were wearing masks at Asian countries’ levels.

Table H. Ratio of Active Cases at the End of 60 Days after Exchanging Mask Wearing Numbers

|  | **country** | **exchanged with** | **Lower limit for 95% CI** | **Ratio** | **Upper limit for 95%**  **CI** |
| --- | --- | --- | --- | --- | --- |
| max | Malaysia | Denmark | 23.86516 | **25.13209** | 26.44875 |
| max | Philippines | Finland | 12.00857 | **13.95786** | 16.19232 |
| max | Taiwan | Norway | 35.53529 | **39.52427** | 43.90114 |
| max | Thailand | Sweden | 33.90308 | **36.76336** | 39.82361 |
| min | Denmark | Malaysia | 0.009502 | **0.041856** | 0.180894 |
| min | Finland | Philippines | 0.00727 | **0.035283** | 0.167807 |
| min | Norway | Taiwan | 0.00432 | **0.025148** | 0.143133 |
| min | Sweden | Thailand | 0.004408 | **0.025332** | 0.142346 |

Next, we present the combined effect of social mobility and NPIs. We provide a combined effect for social mobility and NPIs as it is difficult to estimate the causal analysis for individual variables. We use the Krinsky-Robb method to estimate the combined effect of social mobility and NPIs. After drawing samples of coefficients of social mobility and NPIs, we add the random samples draw and present the mean and confidence interval bounds of these samples as the combined effect and confidence interval bounds of that combined effect.

**S3.3.2 Social Mobility**

Parameter coefficients in Table F show that growth rate increases as mobility increases. If people travel more or move to places with the potential of public gatherings, infected individuals can spread the virus to the susceptible population. Governments, therefore, imposed strict restrictions to reduce mobility. We report the effect of mobility as negative of the coefficients in Table F. Thus, we report the effect of mobility if the mobility numbers were 0 (no mobility change). Results in Fig Z indicates that 0 change in mobility trends (no change in individual mobility trend indicates if people move around as they were before COVID-19) is associated with a daily increase in growth rate by 8.1% (5.6% - 10.6%) as compared to actual cases. Note that a decrease in mobility can also attribute to NPIs. No causality can be claimed on the effect of increased mobility on growth rate.

Fig Z depicts the effect of full mobility across different countries. It shows that even with mask numbers remaining unchanged and NPIs being implemented as they were implemented in that country, increasing mobility can lead to a significant increase in growth rate. Similar to Fig U, we can predict daily active cases with full mobility as shown in Fig AA.


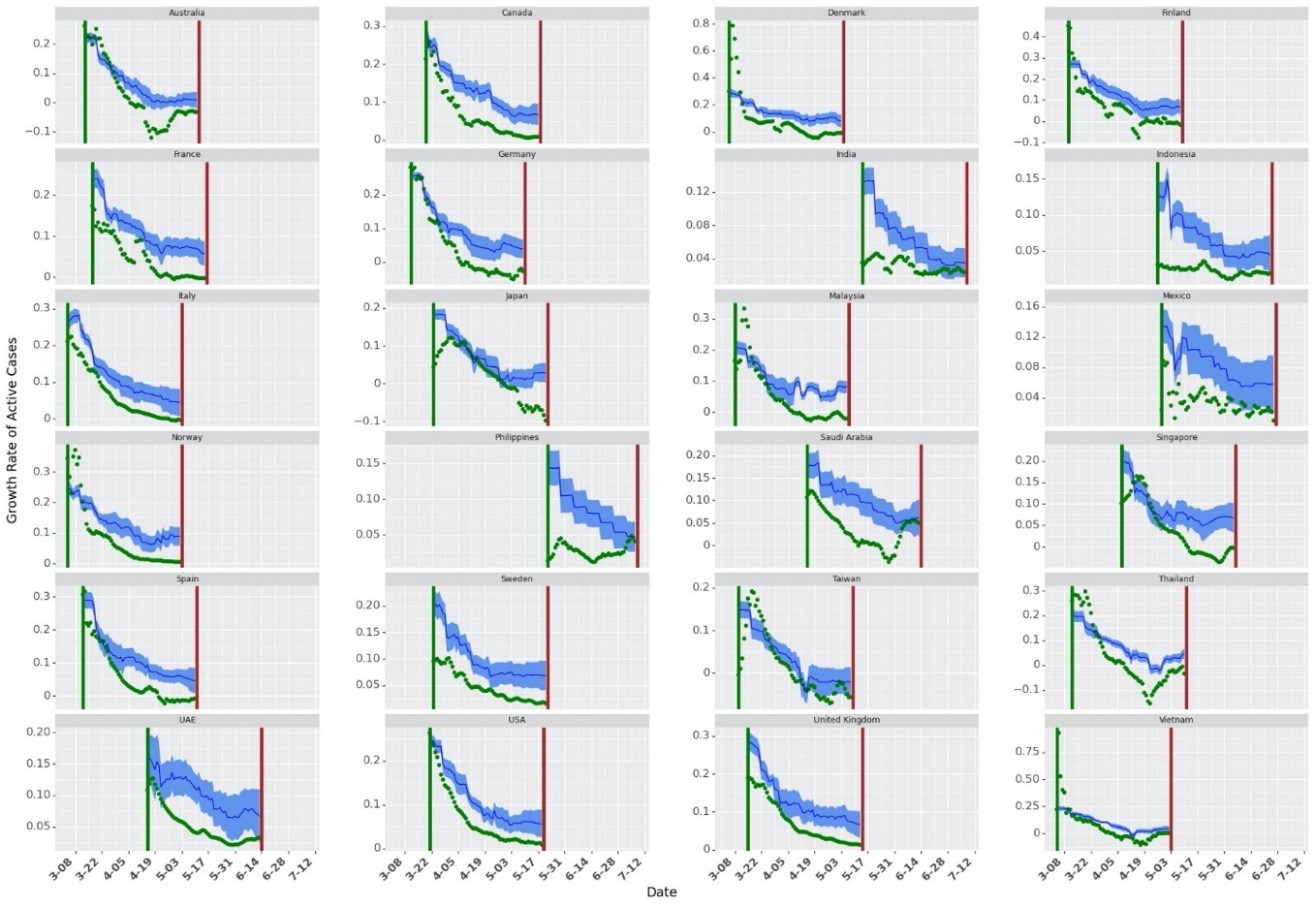


Fig Z. Growth Rate with Full Mobility. The green dots show the actual growth rates across countries. The dark blue line shows the mean of growth rate prediction (for 10000 samples in the Krinksky-Robb method). The blue shaded area shows the confidence bounds around the mean prediction.


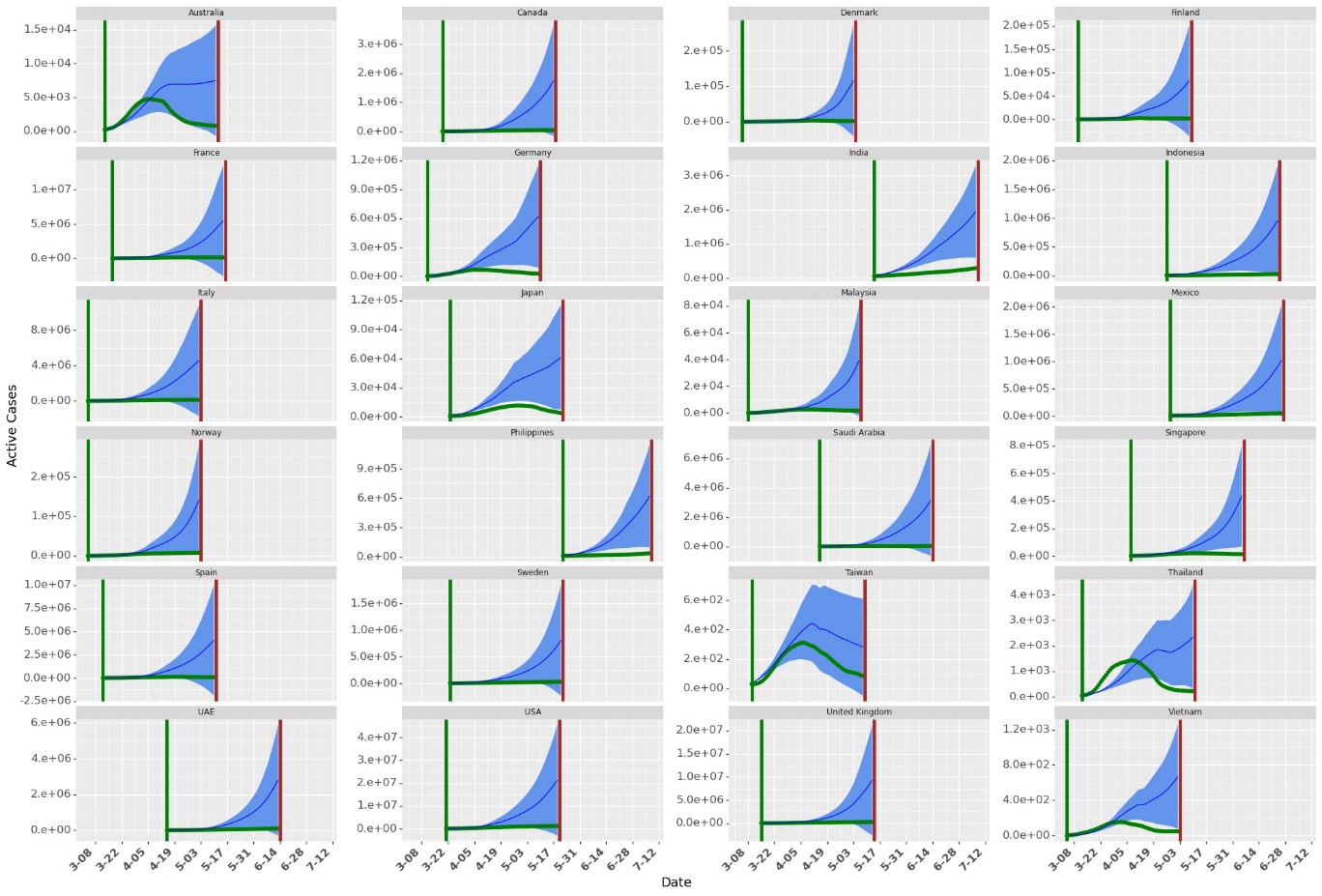


Fig AA. Simulation with no change in Mobility (as compared to pre-COVID-19 mobility). The green dots show the actual daily active cases across countries. The dark blue line shows the mean of active daily cases prediction (for 10000 samples in the Krinksky-Robb method). The blue shaded area shows the confidence bounds around the mean prediction.

Similar to exchanging the mask wearing numbers among the countries with the lowest and highest mask wearing percentages, we simulate the active cases at the end of 60 days by exchanging social mobility among countries with the highest and lowest social mobility. Table I summarizes the results.

Table I. Ratio of Active Cases at the End of 60 Days after Exchanging Social Mobility Numbers

| **Social Mobility** | **Country** | **Exchanged with** | **Lower Limit for 95% confidence Interval** | **Ratio** | **Upper Limit for 95% confidence Interval** |
| --- | --- | --- | --- | --- | --- |
| High | Philippines | Taiwan | 1.81473 | 6.940316 | 26.09045 |
| High | Denmark | United Kingdom | 2.495734 | 2.620681 | 2.750162 |
| High | Sweden | India | 1.163527 | 1.270406 | 1.385541 |
| High | Norway | Spain | 0.998357 | 1.100658 | 1.211927 |
| Low | Spain | Norway | 0.363724 | 0.947547 | 2.438379 |
| Low | India | Sweden | 0.026063 | 0.123776 | 0.576219 |
| Low | United Kingdom | Denmark | 0.314838 | 0.384042 | 0.467267 |
| Low | Taiwan | Philippines | 0.016745 | 0.098078 | 0.561585 |

**S3.3.3 Non-Pharmaceutical Interventions (NPIs)**

Negative and statistically significant estimates for the combined effect of NPIs show that NPIs helped in controlling the spread of the virus. Results in Table F indicate that if mask wearing and mobility remain unchanged, implementing NPIs is associated with a daily drop of infectious cases by 13% (9.2% - 16.2%). Predicted growth rate and daily active cases with no NPIs are shown in Fig AB andFig AC, respectively.


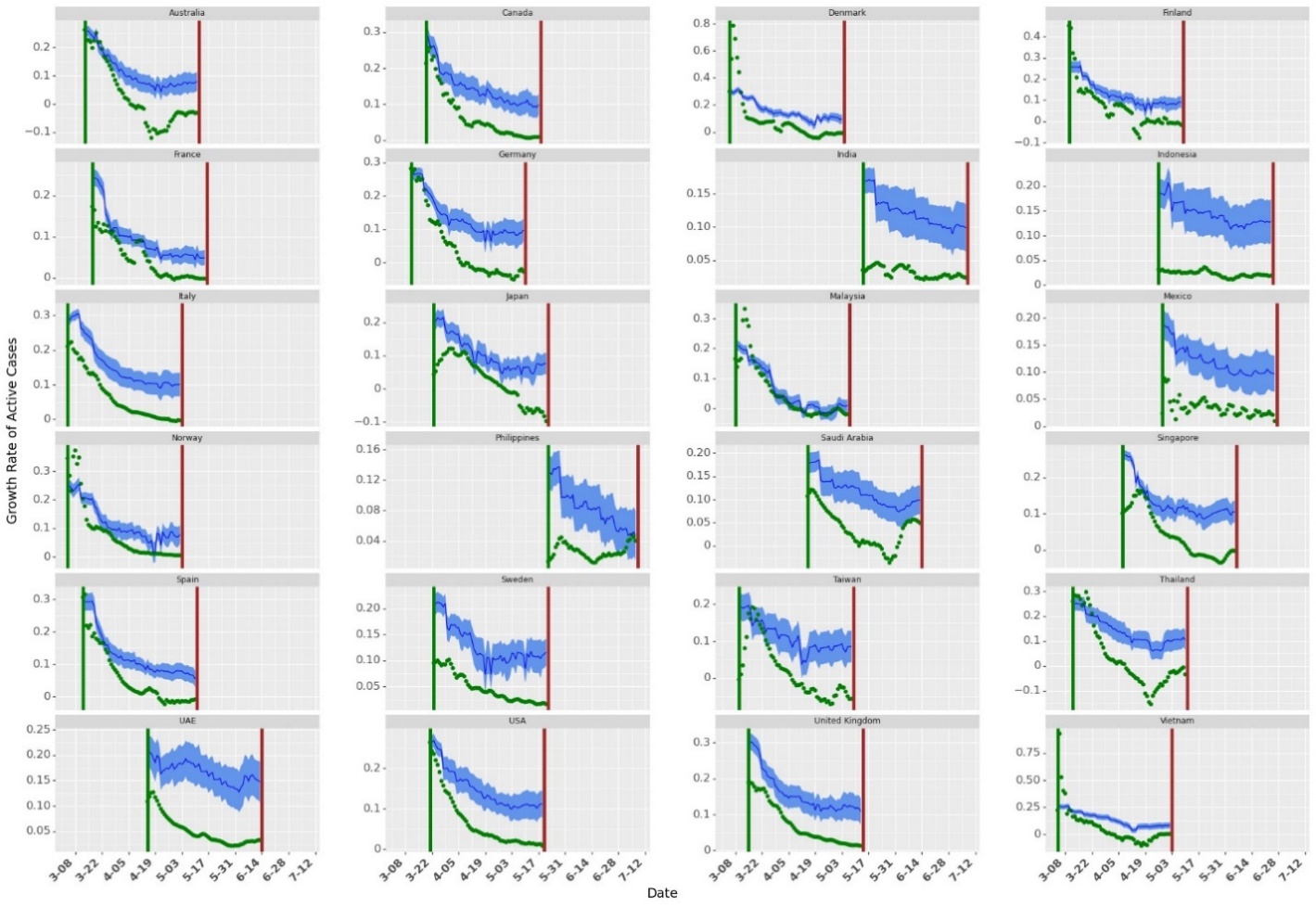


Fig AB. Growth Rate with no NPIs. The green dots show the actual growth rates across countries. The dark blue line shows the mean of growth rate prediction (for 10000 samples in the Krinksky-Robb method). The blue shaded area shows the confidence bounds around the mean prediction.


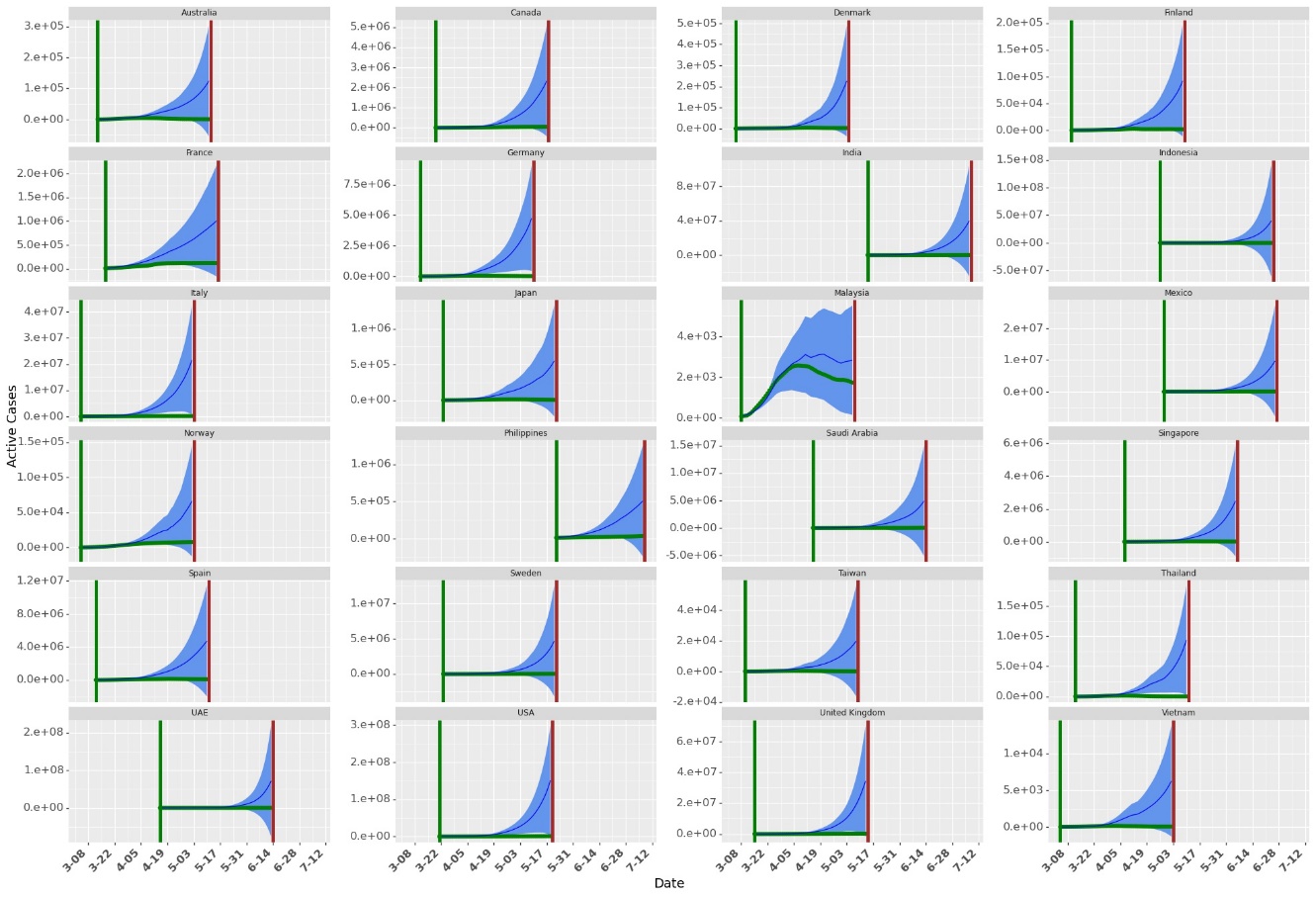


Fig AC. Simulation with no NPIs implemented. The green dots show the actual daily cases across countries. The dark blue line shows the mean of active cases prediction (for 10000 samples in the Krinksky-Robb method). The blue shaded area shows the confidence bounds around the mean prediction.

Similar to exchanging the mask wearing numbers among the countries with the lowest and highest mask wearing percentages, we simulate for the active cases at the end of 60 days by exchanging NPI numbers among countries with the highest and lowest number of NPIs introduced across the country. The results are shown in Table J.

Table J. Ratio of Active Cases at the End of 60 Days after Exchanging NPIsNumbers

| **NPI** | **Country** | **Exchanged with** | **Lower Limit for 95% CI** | **Ratio** | **Upper Limit for 95% CI** |
| --- | --- | --- | --- | --- | --- |
| High | Italy | Finland | 2.551666 | 8.34897 | 26.90575 |
| High | Australia | Norway | 10.81348 | 17.51927 | 28.20859 |
| High | Thailand | Malaysia | 40.38261 | 209.2452 | 1061.602 |
| High | Singapore | France | 10.7138 | 59.20919 | 320.1261 |
| Low | Finland | Italy | 0.12681 | 0.13582 | 0.145342 |
| Low | Norway | Australia | 0.084334 | 0.092976 | 0.102375 |
| Low | Malaysia | Thailand | 0.001354 | 0.006118 | 0.027119 |
| Low | France | Singapore | 0.00753 | 0.015919 | 0.033334 |

**S3.3.4 Combined Effect of Mask, Social Mobility and NPIs**

Similar to presenting the combined effect of mobility and NPIs, we use the Krinsky-Robb method to present the effects for the combined effect of masks, social mobility, and NPIs in the daily drop of growth rate in Fig AD. Fig AD also shows the robustness of the model across different values of $shift$. It also shows the Mean Absolute Percentage Error for 10-fold cross validation used to get the $shift$ that best fit the data. We observe best data fit for a lag of 9 days. Grey vertical lines indicate a shift of 7 days and 11 days. The combined effect of masks, social mobility, and NPIs is estimated to be a 28.1% (24.2%-32%) drop in daily growth rate.

Results show that the effect of masks remain consistent across different transformations and different $shift$. We also observe consistency across different transformations of mask numbers. Furthermore, the total combined effect of masks, social mobility, and NPIs remain consistent as we change $shift$.


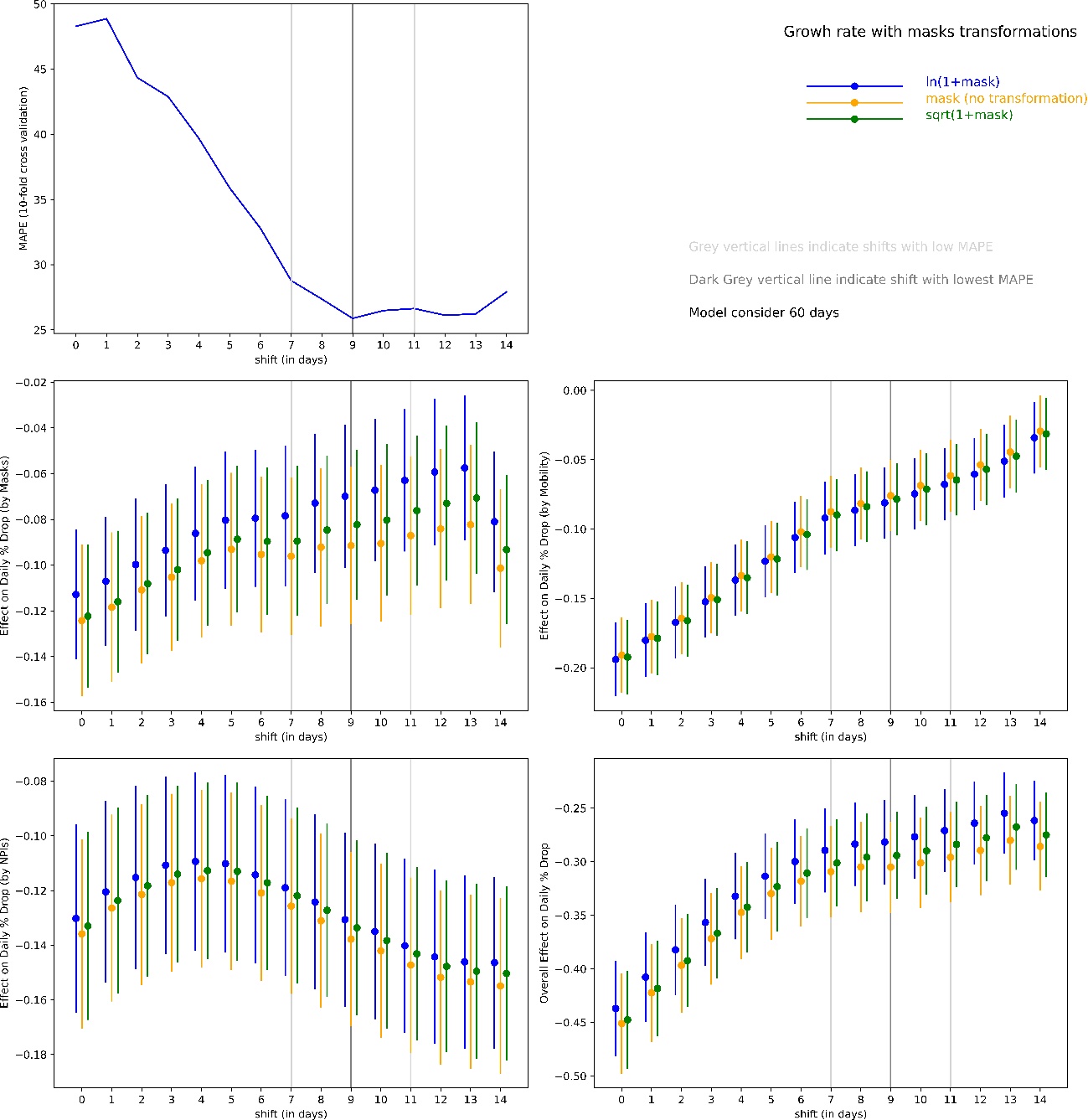


Fig AD. Top left: Mean Absolute Percentage Error under various $shift$ values. Bottom four: Combined effect of Mask, mobility and NPIs in daily % drop of infectious cases under various $shift$ values.

The results show that masks, social mobility, and NPIs lead to significant reduction in total cases, and without these measures, the number of cases could exponentially increase over time.

**S3.4 Testing and Google Trends**

The negative and statistically significant coefficient for testing and Google Trends indicates a daily growth rate drop as these numbers increase. As testing increases, it may show increased daily confirmed cases (as more people get tested and it can discover asymptomatic cases). However, our model uses a lag of 9 days. Thus the cases may not be affected by testing immediately. Thus, a negative coefficient with a lag shows that testing helps achieve a daily drop of growth rate in active infectious cases. Similar to testing, as our model uses a lag of 9 days, increased google trends indicate increased awareness among the citizens regarding COVID-19, leading to more caution against COVID-19.

**S4. Robustness Checks**

**S4.1 Selection of** $th$ **and** $shift$

To filter the initial volatile growth rate, we use a threshold in the model (one for each country). We start collecting data for each country from the day it reaches its threshold. We define threshold as -- the day after which the 7-day average of daily new cases were *th* % of the peak daily new cases observed in that country. Decreasing $th$ will add noise to the model due to high volatility in early growth rate values. However, if the threshold is high, we miss out on important data, particularly during the initial phase when the growth in infections is exponential.

Along with $th$, we also use $shift$ in the growth rate model to capture the delay in the effect of the mask, Non-Pharmaceutical Interventions (NPIs), and mobility. First, we select the value of $th$ and then use that $th$ to find optimal $shift$ for our analysis. We calculate log-likelihood for growth rate model under different $th\in[0.01, 0.02,\ldots, 0.3]$. For each $th$, we run the model for different $shift$. Using multiple $lag$ ensures that the model performance is consistent with different values of $shift$. It also ensures that we do not select $th$ that performs well by chance. We select $th$ based on maximum average log-likelihood for different $shift$. We start with a threshold value of 0.01 and start increasing it. Fig AE shows that the average log-likelihood for growth rate does not change much after $th=0.18.$ We use a $th=0.20$ in the rest of the paper. We use a sensitivity test to check the consistency of the model.


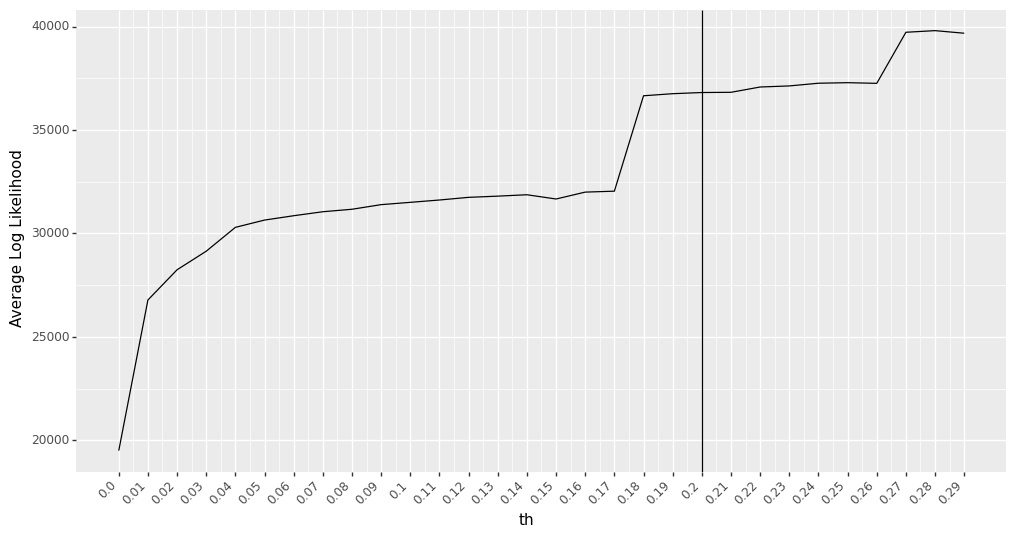


Fig AE. Average Log-likelihood Values for Different $th$. The model performs the best (based on maximum likelihood function) when $th=0.28$. However, the performance change is not significant after $th=0.18$. So, we use $th=0.2$ in our analysis in this research.

In the growth rate model, we use $shift$ to estimate the parameter coefficients as shown in Equation S8. After selecting $th$, we use cross validation to find the best $lag$ that fits the data. We use 10-fold cross-validation with Mean Average Percentage Error (MAPE) as a metric for out-of-sample data points to select $shift$with the best fit. Fig AF shows the average MAPE (for 10-fold cross validation) for different lags. $shift=9$ days shows the best fit with MAPE’s minimum value.


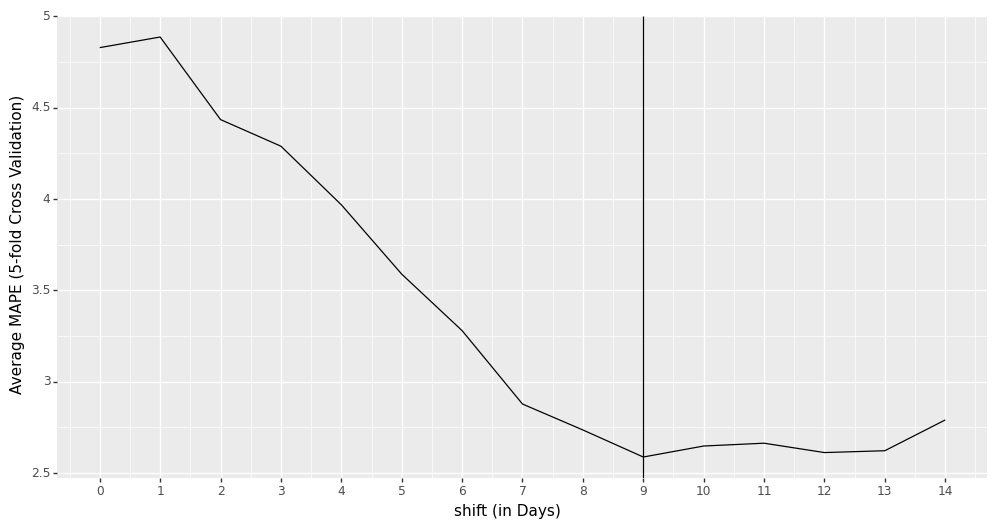


Fig AF. Average MAPE from 10-fold Cross Validation Different $lag$. Minimum value for Mean Absolute Percentage Error (MAPE) is obtained at a $lag=9 days$.

**S4.2 Model Estimation Sensitivity to** $th$ **and** $shift$

We use sensitivity test to check the consistency of the parameter estimates for different values of $th$ and $shift$. The performance of the model remains consistent on changing the values of $th$ from 0.18 to 0.22 as shown from parameter estimates in Fig AG (we use a $shift$ of 9 days). The performance of the model remains consistent on changing the values of $shift$ from 7 days to 11 days as shown from parameter estimates in Fig AH (we use $th=0.2$). We transform masks as $\ln\left( 1+mask_{j,t} \right).$


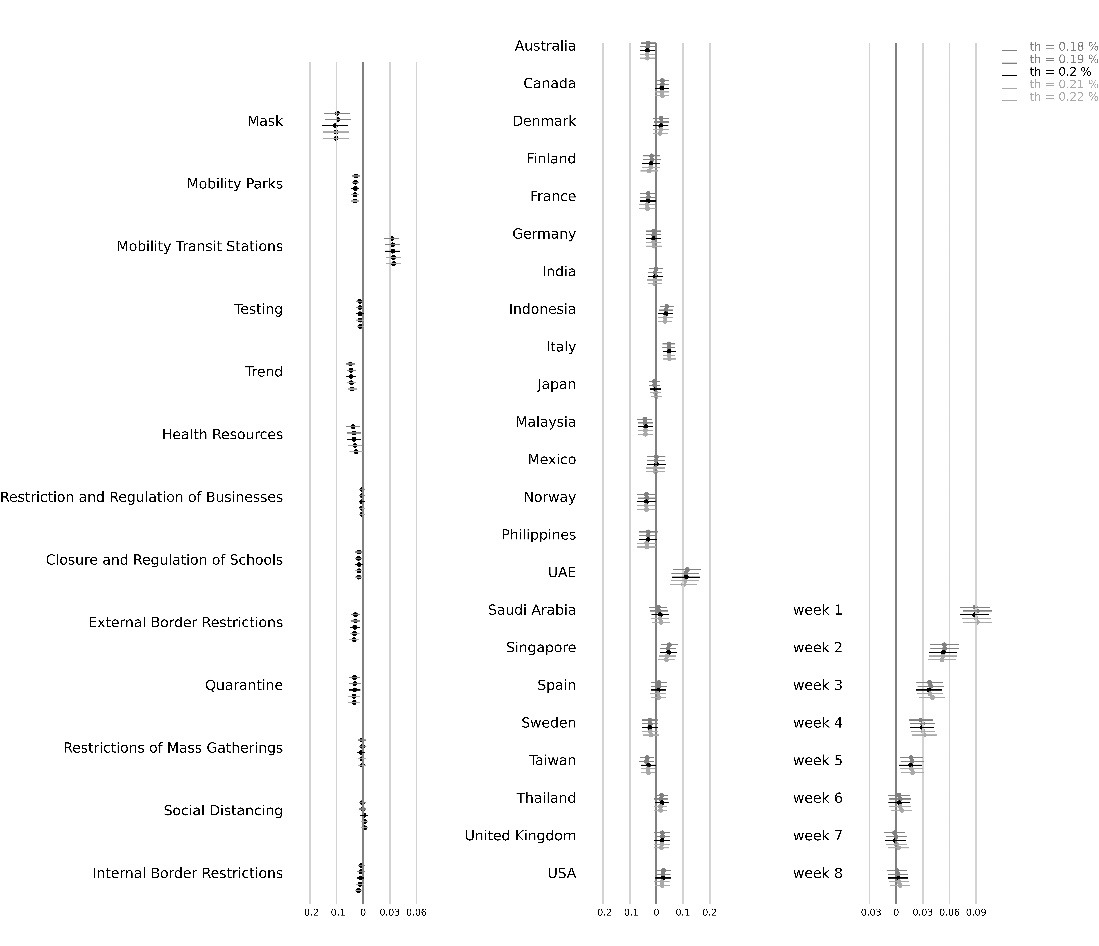


Fig AG. Parameter Estimates for Growth Rate Model for Different $th$. Horizontal lines represent the upper and lower confidence interval bound for the parameter estimates. We show the results for a lag of 9 days with $th\in[0.18, 0.19, 0.2, 0.21, 0.22]$. The results indicate that the model is robust to different lags as the parameter estimates show consistency. Vietnam has been kept as the base country (the fixed country effect is 0).


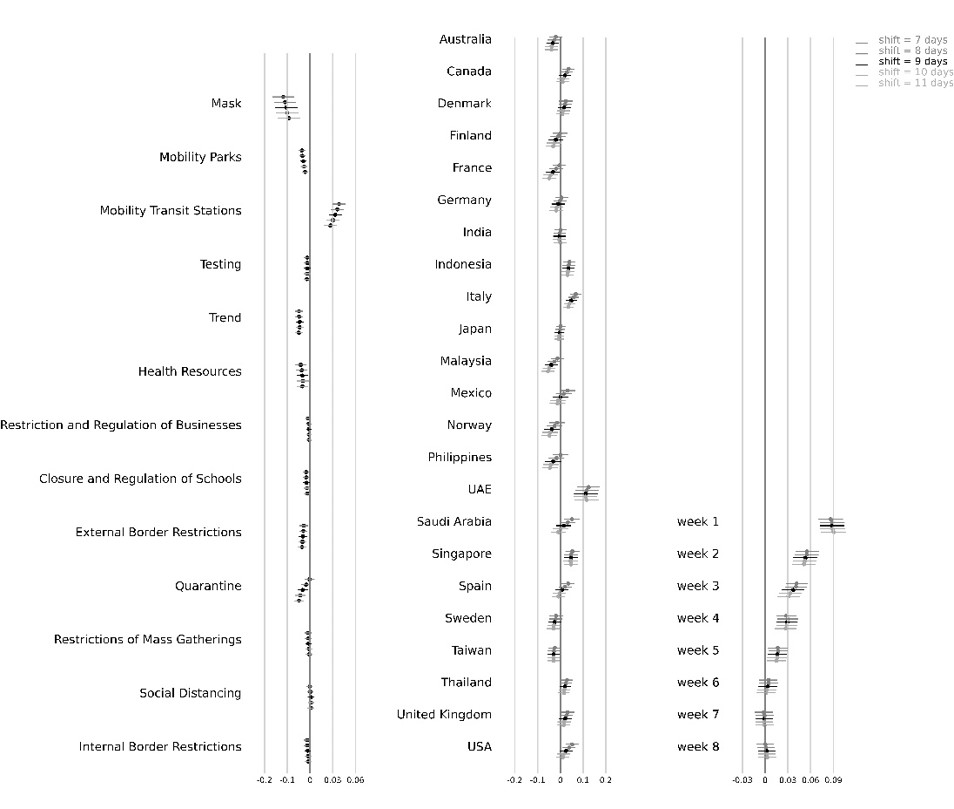


Fig AH. Parameter Estimates for Growth Rate Model for Different $shift$. Horizontal lines represent the upper and lower confidence interval bound for the parameter estimates. We show the results for a lag of 7 days to 11 days with $th=0.2$. The results indicate that the model is robust to different $shift$ as the parameter estimates show consistency. Vietnam has been kept as the base country (the fixed country effect is 0).

**S4.3 Handling Data Error in Active Cases**

In Fig E, we observe data reporting issues in Norway, Sweden, and United Kingdom. The recovered cases are reported late in Norway, not reported in Sweden, and recorded very low in the United Kingdom. We use data from Johns Hopkins Resource Center. Our analysis uses total confirmed cases to find active cases for these three countries. However, this may lead to bias in the results. To check the bias, we run the model without these three countries. Fig AI shows the parameter estimates after excluding these countries. In Fig AI, we show the parameter coefficients when $shift=9$ days and $th=0.2$.

The results show that the model parameter estimates are robust to excluding Norway, Sweden, and the United Kingdom from the model. However, the model slightly overestimates the coefficient of masks after excluding Norway, Sweden, and United Kingdom (compared to the growth model with data from all the 24 countries). All three countries are in Europe, where wearing face masks is not as common as in Asian countries. Moreover, Norway, and Sweden (along with Denmark and Finland) have the lowest percentage of people who wear face masks in public in our data set.


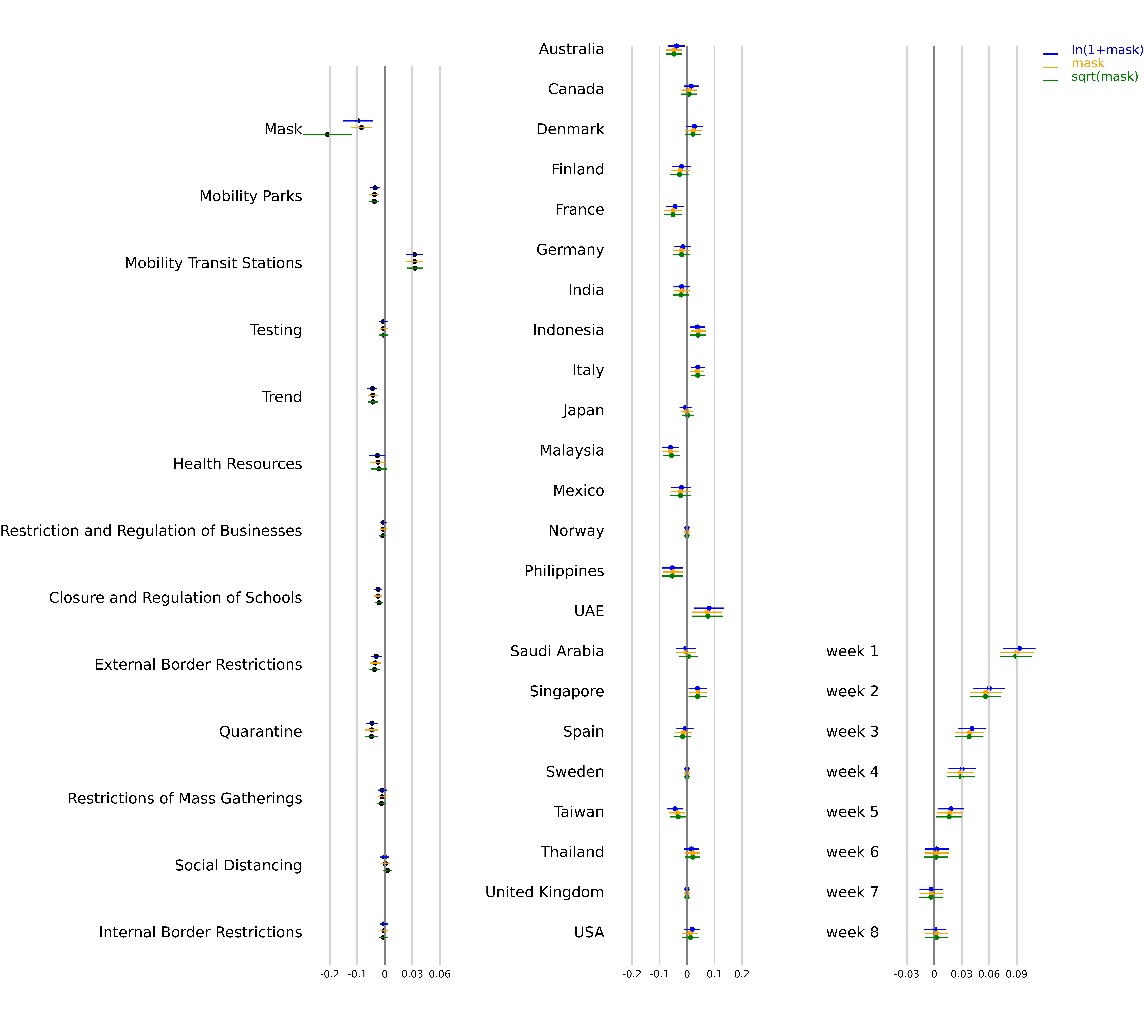


Fig AI. Parameter Estimates for Growth Rate Model after excluding Norway, Sweden and United Kingdom from the analysis. Horizontal lines represent the upper and lower confidence interval bound for the parameter estimates. We show the results for different transformations of $mask_{j,t}$. The results indicate that the model is robust to different transformations as the parameter estimates show consistency. Vietnam has been kept as the base country (the fixed country effect is 0)

**S4.4 Robustness Check for Mobility**

In our analysis, we used Google’s community mobility reports numbers as a measure of social mobility as android operating devices are more common than iOS, particularly in Asian countries [14]. Also, Apple’s Community Mobility Reports record data only when an individual opens Apple Maps. To check the robustness of the combined effect of masks, social mobility, and NPIs, we also consider Apple’s community mobility numbers as a measure of social mobility. Apple released data for change in trends for driving and walking for all the 24 countries considered in this work. Fig AJ shows the combined effect after substituting with Apple’s mobility report. Consistency of the results show that the estimates for the model are robust.


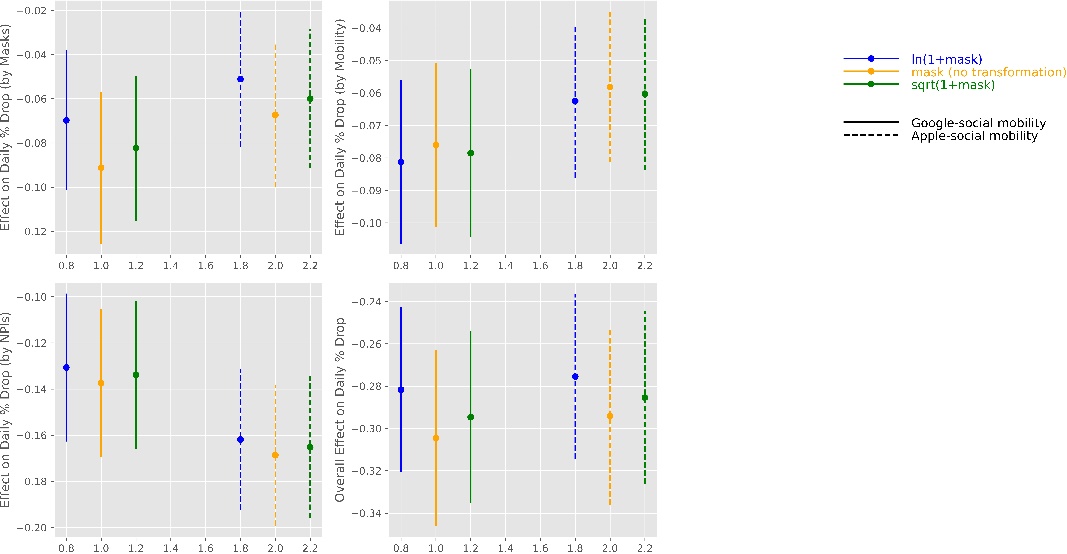


Fig AJ. Combined effect of masks, social mobility, and NPIs with mobility numbers from Google and Apple.

**S4.5 Alternative Specifications**

We build two robustness models to check the consistency and reliability of the parameter estimates of the growth rate model. In the first robustness check, we use exponential smoothing as shown in Equations S9-S10. In the second model, we use a control function approach to identify the impact of masks on the spread of COVID-19. We discuss it in detail in this subsection.

**S4.5.1 Model 1: Exponentially Smoothed Variates for Growth Rate**

In the first specification, we use exponential smoothing to estimate masks, NPIs, and social mobility parameters. In our base model in Equation S8, the growth rate is defined as a function of masks, NPIs, social mobility, trend, and testing at a lag of $shift$ days. On any day $t$, this model ignores the value of the variates from days $t-shift+1$ to $t$ (discussed in Fig A). In this model, we do not ignore variates between $t-shift$ and $t$ and use exponential smoothing average to check the robustness of our model. To check the consistency of the parameter estimates for different transformations of masks, mobility, NPIs and the combined effect of masks, social mobility and NPIs is shown in Fig AK.

We also show the parameter estimates from the growth model for comparison. The results show that the parameter estimates for both models are close and consistent, thus showing the robustness of the results in Table F.


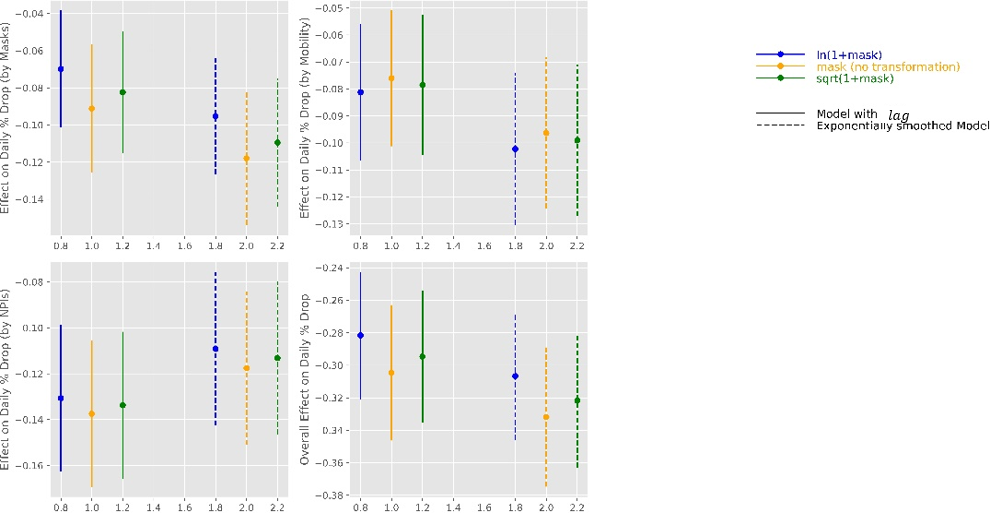


Fig AK. Effect on daily % drop of growth rate by Masks (top left), Mobility (top right), NPIs (bottom left) and the Combined effect of masks, social mobility and NPIs (bottom right) with $shift=9$ days for exponentially smoothed model.

In the next model, we use a control function approach to check the robustness of our estimates in Table F. The control function approach considers an error variable based on an exogenous variable that is not correlated with the response variable but with an instrumental variable. We use the number of deaths per thousand people for SARS, H1N1 and MERS CoV as our exogenous variables.

**S4.5.2 Model 2: Control function Approach to Growth Rate**

In the control function approach, we first predict the average value of $mask_{j,t}$ by using the number of deaths per thousand people in each country by SARS, MERS-CoV, and H1N1. Results from predicting masks using disease per thousand people are shown in Table K and the parameter estimates are shown in Table L.

We consider all the available data sets (from February 21, 2020, to July 8, 2020) to estimate SARS, H1N1, and MERS coefficients. We convert the numbers for deaths due to SARS, H1N1, and MERS into a binary variable (1 if the number for a country is greater than the median).

Table K. Results Statistics for Predicting $log\left( 1+mask \right)$ using SARS, H1N1 and MERS

| R-squared: | 0.056 |
| --- | --- |
| Adj. R-squared: | 0.055 |
| F-statistic: | 65.24 |
| Probability (F-statistic): | 0 |
| Log-Likelihood: | -8108 |
| AIC: | 16200 |
| BIC: | 16250 |
| No. Observations: | 3312 |
| Degree of Freedom: Residuals: | 3308 |
| Degree of Freedom: Model: | 3 |

Table L. Results from Predicting $log\left( 1+mask \right)$ using SARS, H1N1 and MERS

| coef | **std** | **err** | **t** | **P>\|t\|** | **[0.025** | **0.975]** |
| --- | --- | --- | --- | --- | --- | --- |
| Sars | 0.5013 | 0.179 | 2.797 | 0.005 | 0.15 | 0.853 |
| H1N1 | -1.3904 | 0.101 | -13.78 | 0 | -1.588 | -1.193 |
| Mers | 0.1556 | 0.114 | 1.363 | 0.173 | -0.068 | 0.38 |
| const | 6.9685 | 0.071 | 98.706 | 0 | 6.83 | 7.107 |

We use $mask_{j,t}$ along with the residuals from prediction model, $e=mask_{j,t}-\hat{mask_{j}}$, as control function in Equation S11. Fig AL illustrates the results for the combined effect of masks, social mobility, and NPIs. We use a $shift$ of 9 days. We also show the combined effect of masks, social mobility, and NPIs without control function (the focal model in this paper) to show that the combined effect of masks, social mobility, and NPIs estimated in Fig AD are not appreciably different.


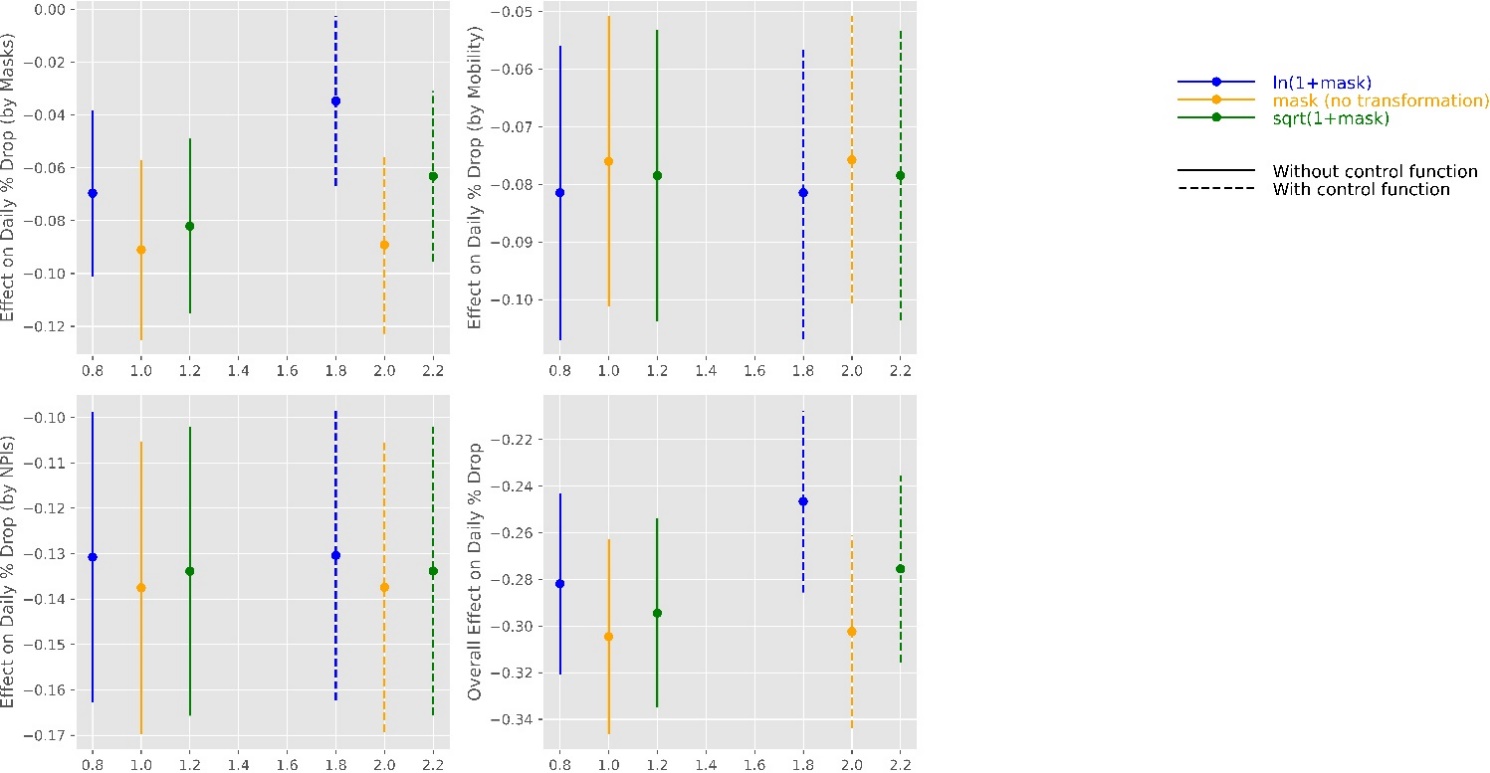


Fig AL. Combined effect of Mask, Social Mobility, and NPIs with and without control functions. We use growth rate model with a $shift$ of 9 days.

**S4.5.3 Lasso Regression**

Governments worldwide introduced NPIs to enforce social distancing through policies like quarantine, restriction on mass gatherings or closure of schools and businesses. NPIs led to decreased social mobility. For example, there were no major gatherings in railway or bus stations as rails and buses were closed down. In our analysis, NPIs and social mobility across different location types are correlated. This may lead to multicollinearity that may lead to unstable coefficients. We use penalized linear regression (Lasso regression) as a robustness check for shrinking the coefficients of highly correlated variates.

Lasso regression can also handle multicollinearity in the data as it shrinks the coefficients to 0 using L1-norm. Lasso regression pushes the coefficients of insignificant variables to 0, thereby introducing sparsity in the model. Our analysis show that the Lasso regression pushes the coefficients for all social mobility indicators to zero except mobility in parks and transit stations. As we observe the correlation between different indicators of social mobility in Table B, Lasso regression provides validation for the selection of two (out of 6) indicators of mobility. Fig AM shows the coefficients for the Lasso growth model. Equation S15 represents a Lasso regression model where $n$ is the sample size, $\beta$ is a vector of coefficients, $X$ is input variables, and $Y$ is the outcome variable. We use 5-fold cross-validation to find $\lambda$ that best fits the out-of-sample test data.

$$\beta= arg\min_{\beta\in R^{p}} \left\{ \frac{\left( Y-X\beta\right)^{2}}{n} + \lambda\left| \left| \beta\right| \right|_{1} \right\} (S15)$$

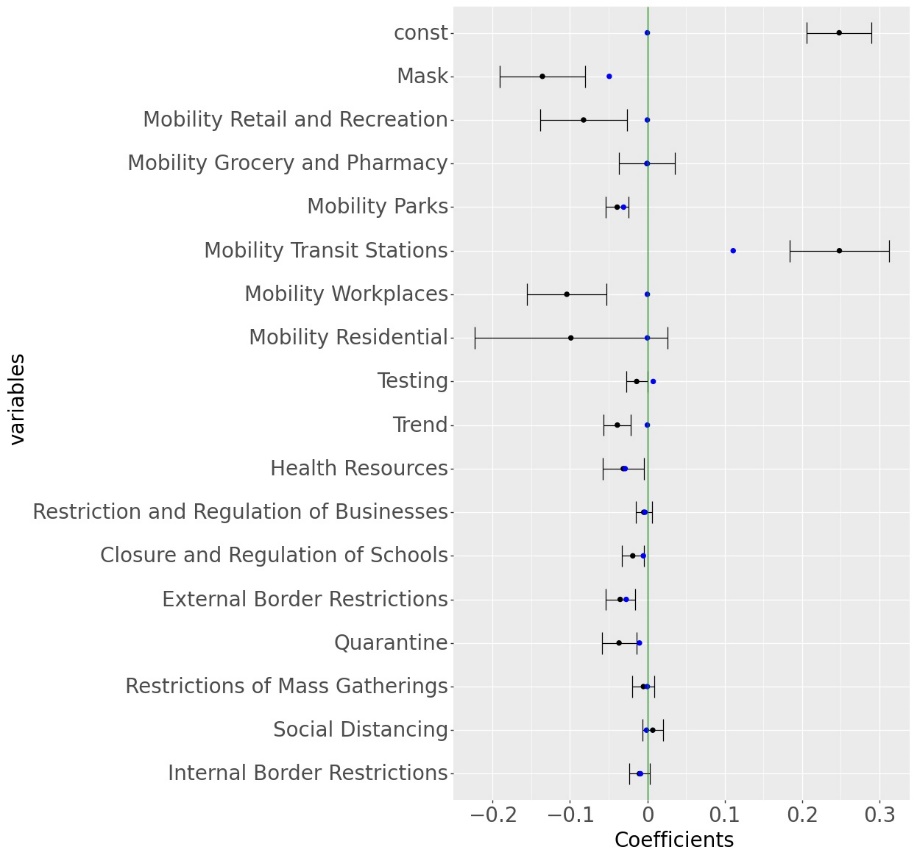


Fig AM. Parameter Estimation from Ordinary Least Square and Lasso Regression Model for growth rate. The blue dot represents the coefficients estimated from the Linear Regression model. The error bars represent the upper and lower confidence interval for the coefficients obtained from Ordinary Least Squares. The blue dot represents the coefficients estimated from the Lasso Regression model.

**S4.6 Selecting Period of Analysis**

To filter out initial volatile growth rates during the start of the pandemic, we use a threshold $th$ as discussed before. We collect data for up to 60 days for a country, from the day it reaches $th$ percent of peak daily cases in that country. However, the model estimates could be biased and fit the given set of data points. To estimate the robustness of the model, we estimate the model parameters by collecting data for up to $D$ days from the day that country reaches threshold $th$. The results for the combined effect of mask, social mobility and NPIs for different $D\in[35,45,55,65,75,85]$ is shown in Fig AN.


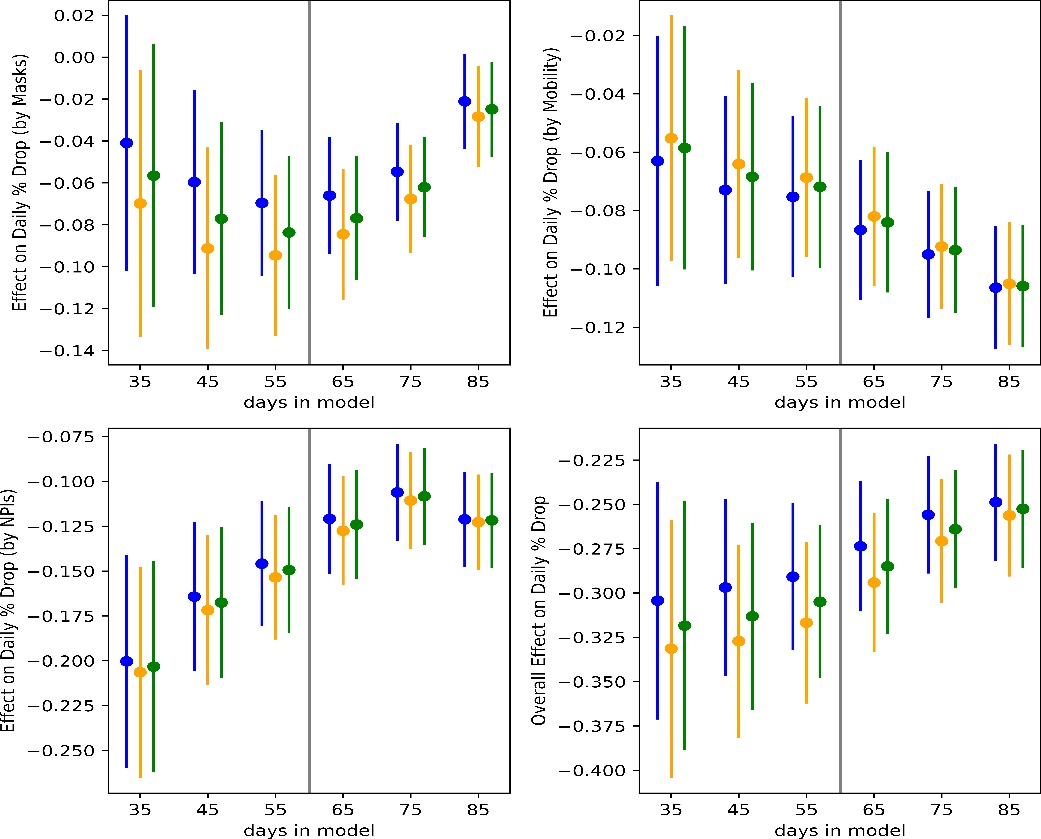


Fig AN. Combined effect of Mask, Social Mobility and NPIs when data is collected for different number of days. We consider a shift of 9 days for these results (Note that a shift of 9 days was the best fit for a model that used data for 60 days. The results show consistency within the bounds of the combined effects.

**S4.7 Interpolating Mask Survey Numbers Between Survey Days**

We use survey data released by the Institute of Global Health Innovation (IGHI) at Imperial College London and YouGov4 for reported mask-wearing across multiple countries. The data present global insights on people’s reported behavior in response to COVID-19. The dataset provides the percentage of the population in each country who report wearing a mask in public places. Because these surveys were conducted at an interval of several days, we used linear interpolation to estimate the percentage of the population that would wear masks in public spaces for days when the data were unavailable (Fig D). To check the robustness of estimates from the model, we use a quadratic interpolation method to estimate the percentage of the population that would wear masks in public spaces for days between surveys. The estimate for stated mask wearing using quadratic interpolation is shown in Fig AO.


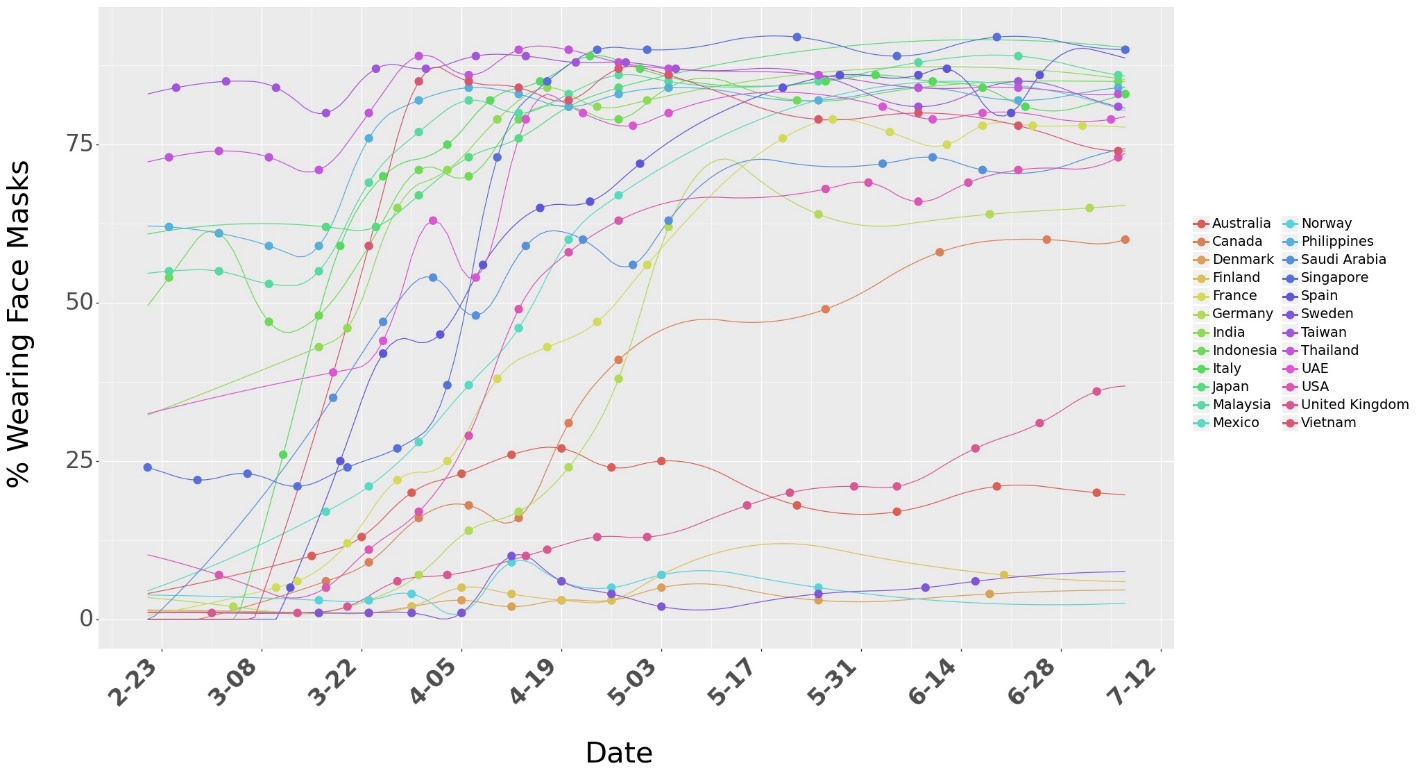


Fig AO. Survey data on percentage of people who say they wear a face mask when in public spaces. We use quadratic interpolation to consider mask numbers for days between surveys days. The dots represent the raw numbers from surveys.

Fig AP shows the results for the association of mask, social mobility, NPIs, and the combined effect of masks, social mobility, and NPIs on the growth rate for quadratic interpolation. We use a $shift$ of 9 days and transformed masks as our focal model ($ln(1+mask)$). We also show results for linear interpolation (the focal model in this paper) to show that the parameter estimates are not appreciably different.

**
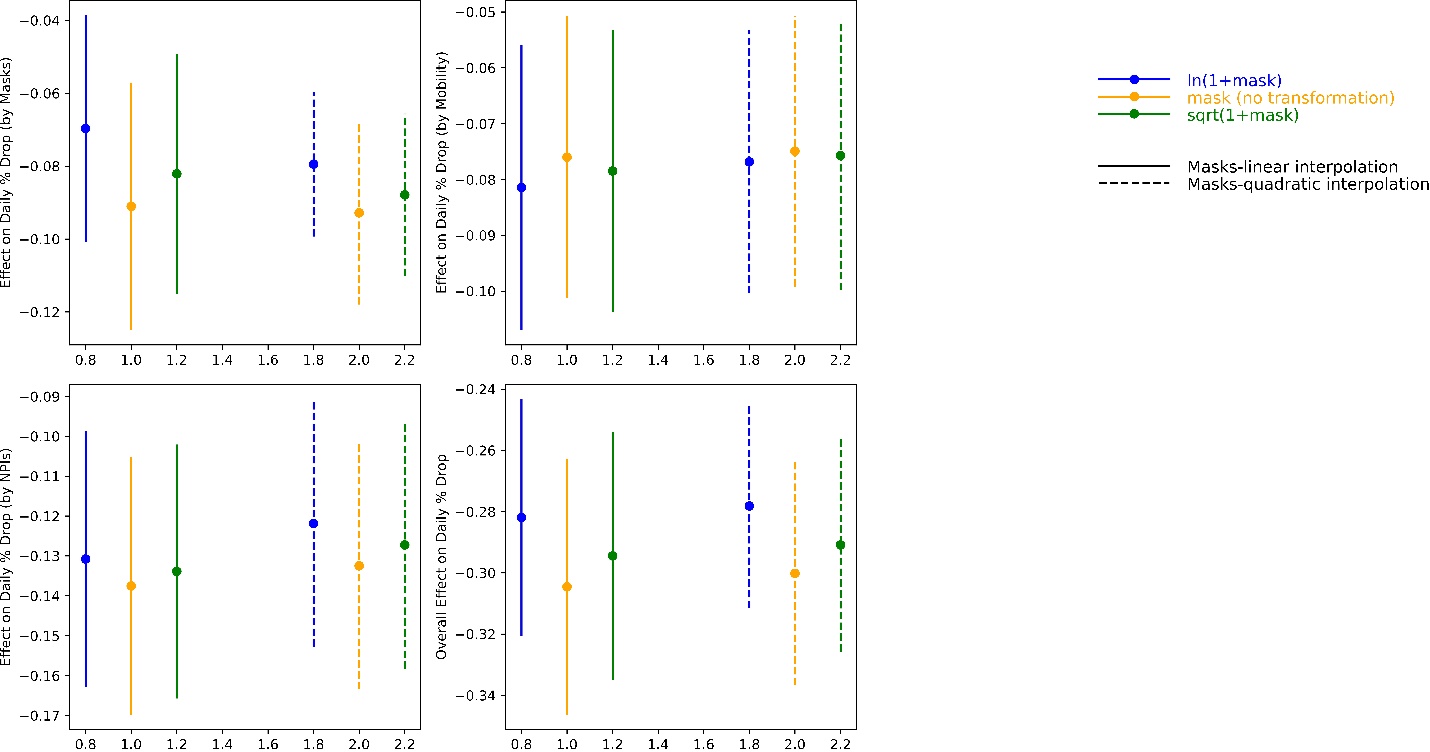
**

Fig AP. Combined effect of Mask, Social Mobility and NPIs under different interpolation for mask survey numbers. We use growth rate model with a $shift$ of 9 days and consider data for 60 days.

**References**

1. Hsiang S, Allen D, Annan-Phan S, Bell K, Bolliger I, Chong T, Druckenmiller H, Huang LY, Hultgren A, Krasovich E, Lau P. The effect of large-scale anti-contagion policies on the COVID-19 pandemic. Nature. 2020 Aug;584(7820):262-7.
2. Lauer SA, Grantz KH, Bi Q, Jones FK, Zheng Q, Meredith HR, Azman AS, Reich NG, Lessler J. The incubation period of coronavirus disease 2019 (COVID-19) from publicly reported confirmed cases: estimation and application. Annals of internal medicine. 2020 May 5;172(9):577-82.
3. Beglin CK. Why Was Mask Wearing Popular In Asia Even Before Covid-19? Psychology Today, 2020 May 17 [Cited 2020 August 28]. Available from: <https://www.psychologytoday.com/us/blog/culture-shocked/202005/why-was-mask-wearing-popular-in-asia-even-covid-19>
4. Yang J. A quick history of why Asians wear surgical masks in public. Quartz. 2014 November 19 [Cited 2020 August 28]. Available from: https://qz.com/299003/a-quick-history-of-why-asians-wear-surgical-masks-in-public/
5. Personal measures taken to avoid COVID-19. [Cited 2020 September 9]. Available from: <https://yougov.co.uk/topics/international/articles-reports/2020/03/17/personal-measures-taken-avoid-covid-19>
6. COVID-19 Data Repository by the Center for Systems Science and Engineering (CSSE) at Johns Hopkins University. [Cited 2020 July 15]. Available from: https://github.com/CSSEGISandData/COVID-19
7. COVID-19 Community Mobility Reports. [Cited 2020 September 9]. Available from: <https://www.google.com/covid19/mobility/>
8. Mobility Trends Reports. [Cited 2020 July 21]. Available from: <https://covid19.apple.com/mobility>
9. Morath E. How Many U.S. Workers Have Lost Jobs During Coronavirus Pandemic? There Are Several Ways to Count. WSJ. 2020 June 3 [Cited 2020 August 14]. Available from: https://www.wsj.com/articles/how-many-u-s-workers-have-lost-jobs-during-coronavirus-pandemic-there-are-several-ways-to-count-11591176601
10. Kochhar R. Hispanic women, immigrants, young adults, those with less education hit hardest by COVID-19 job losses. Pew Research Center. 2020 June 9 [Cited 2020 July 10]. Available from: <https://www.pewresearch.org/fact-tank/2020/06/09/hispanic-women-immigrants-young-adults-those-with-less-education-hit-hardest-by-covid-19-job-losses/>
11. Guina R. Coronavirus Layoffs - Job Losses And Furloughs Are Even Impacting ‘Safe’ Jobs. Forbes. 2020 May 20 [Cited 2020 August 10]. Available from: https://www.forbes.com/sites/ryanguina/2020/05/05/coronavirus-layoffs-are-impacting-safe-jobs/?sh=3d8926613e17
12. Cheng C, Barceló J, Hartnett AS, Kubinec R, Messerschmidt L. COVID-19 government response event dataset (CoronaNet v. 1.0). Nature human behaviour. 2020 Jul;4(7):756-68.
13. Capron M. How many coronavirus cases are asymptomatic? CDC and other data range as high as 50%. Miami Herald. 2020 April 5 [Cited 2020 August 28]. Available from: <https://www.miamiherald.com/news/coronavirus/article241703806.html>
14. Ritchie H, Mathieu E, Rodés-Guirao L, Appel C, Giattino C, Ortiz-Ospina E, et al. (2020) - Coronavirus (COVID-19) Testing. *Published online at OurWorldInData.org.* [Cited 2020 July 18]. Available from: <https://ourworldindata.org/coronavirus-testing>
15. Explore what the world is searching. [Cited 2020 July 14]. Available from: https://trends.google.com/trends/?geo=US
